# *cis*-regulatory variation modulates susceptibility to enteric infection in the *Drosophila* genetic reference panel

**DOI:** 10.1186/s13059-019-1912-z

**Published:** 2020-01-17

**Authors:** Michael V. Frochaux, Maroun Bou Sleiman, Vincent Gardeux, Riccardo Dainese, Brian Hollis, Maria Litovchenko, Virginie S. Braman, Tommaso Andreani, Dani Osman, Bart Deplancke

**Affiliations:** 1 Laboratory of Systems Biology and Genetics, Institute of Bioengineering, Ecole Polytechnique Fédérale de Lausanne (EPFL) and Swiss Institute of Bioinformatics, Lausanne, Switzerland; 2 Current Address: Laboratory of Integrative Systems Physiology, Institute of Bioengineering, Ecole Polytechnique Fédérale de Lausanne (EPFL), Lausanne, Switzerland; 3 Current Address: Department of Biological Sciences, University of South Carolina, Columbia, South Carolina USA; 4 Laboratory of Systems Biology and Genetics, Institute of Bioengineering, Ecole Polytechnique Fédérale de Lausanne (EPFL), Lausanne, Switzerland; 5 Computational Biology and Data Mining Group, Institute of Molecular Biology, Johannes Gutenberg-Universität Mainz, Mainz, Germany; 6 Faculty of Sciences III and Azm Center for Research in Biotechnology and its Applications, LBA3B, EDST, Lebanese University, Tripoli, 1300 Lebanon

## Abstract

**Background:**

Resistance to enteric pathogens is a complex trait at the crossroads of multiple biological processes. We have previously shown in the *Drosophila* Genetic Reference Panel (DGRP) that resistance to infection is highly heritable, but our understanding of how the effects of genetic variants affect different molecular mechanisms to determine gut immunocompetence is still limited.

**Results:**

To address this, we perform a systems genetics analysis of the gut transcriptomes from 38 DGRP lines that were orally infected with *Pseudomonas entomophila*. We identify a large number of condition-specific, expression quantitative trait loci (*local*-eQTLs) with infection-specific ones located in regions enriched for FOX transcription factor motifs. By assessing the allelic imbalance in the transcriptomes of 19 F1 hybrid lines from a large round robin design, we independently attribute a robust *cis*-regulatory effect to only 10% of these detected *local*-eQTLs. However, additional analyses indicate that many *local*-eQTLs may act in *trans* instead. Comparison of the transcriptomes of DGRP lines that were either susceptible or resistant to *Pseudomonas entomophila* infection reveals *nutcracker* as the only differentially expressed gene. Interestingly, we find that *nutcracker* is linked to infection-specific eQTLs that correlate with its expression level and to enteric infection susceptibility. Further regulatory analysis reveals one particular eQTL that significantly decreases the binding affinity for the repressor Broad, driving differential allele-specific *nutcracker* expression.

**Conclusions:**

Our collective findings point to a large number of infection-specific *cis-* and *trans-*acting eQTLs in the DGRP, including one common non-coding variant that lowers enteric infection susceptibility.

## Background

Deciphering the relationship between genomic and phenotypic variation is a central goal in genetics. Genome-wide association studies (GWAS) have been extensively used to address this challenge by looking for variants that could explain a certain fraction of the genetic variance of phenotypes [1, 2]. More often than not, those variants are located in non-coding regions of the genome, rendering the inference of their putative function difficult [3–6]. Therefore, the study of intermediate molecular traits, such as gene expression levels, and how they are affected by genomic variation is a powerful complementary approach to linking geno- to phenotype [7, 8].

Since the first expression quantitative trait locus (eQTL) report in yeast [9], it has become clear that eQTLs could account for a substantial proportion of variability in gene expression following a cellular or organismal response to external stimuli. These eQTLs in turn advanced our understanding of the genetic basis of disease susceptibility. Indeed, eQTL studies in both mouse and human using monocytes, macrophages, dendritic cells, or other immune cells have been useful to better understand how genetic regulatory effects affect autoimmune disease [10–12], inflammatory bowel disease [13], resistance to *Salmonella* [14], and the molecular response to an infection stimulus [15–18]. These advances motivated the establishment of even larger-scale projects such as DICE (Database of Immune Cell Expression, eQTL, and Epigenomics) to characterize gene expression in all human immune cell types and to study how genetic variants affect these immune cell-related transcriptomes [19]. However, eQTL-related studies aimed at better understanding the genetic and molecular basis underlying gut immunocompetence have been lacking for practical and ethical reasons. Indeed, human intestine eQTL studies have to our knowledge so far been restricted to inflammatory bowel disease [13, 20–23].

A valuable alternative model to uncover the genetic and molecular mechanisms underlying variation in gut immunocompetence is *Drosophila melanogaster* given that this organism is by now widely used to study the biological processes mediating the response to enteric infection [24–28]. Moreover, previous work including ours has shown that gut immunocompetence is a highly variable and heritable trait, not only in human [29] and mouse [30], but also in *Drosophila* [31, 32]. Consequently, population resources such as the *Drosophila* Genetic Reference Panel (DGRP) can be effectively used to study the molecular nature of enteric infection-induced gene expression variation. In this study, we therefore explored the effect of genetic variation on gene expression and organismal phenotypes in the context of in vivo enteric infection in the DGRP. Despite several valuable eQTL studies in *Drosophila* involving the DGRP [33–37] and the *Drosophila* Synthetic Population Resource (DSPR) [38–40], none have so far focused on the response to infection.

To do so, we generated a large set of *Drosophila* control and *Pseudomonas entomophila* (*P.e*.)-infected gut transcriptomes to systematically investigate the link between gut gene expression levels and genetic variation. We used *P.e.* because it is a severe pathogen [41] that, along with other *Pseudomonas* species, is a natural pathogen to the fly [42]. We showed that genotype is a major determinant of global gene expression levels, revealing a large number of both shared and condition-specific *local-*eQTLs [43–45]. We then validated and catalogued these *local-*eQTLs into *cis* and *trans-*acting eQTLs using allele-specific expression on a set of F1 siblings from crosses between isogenic DGRP lines. Importantly, we identified *nutcracker* (*ntc*) as a gene that is differentially expressed between susceptible and resistant DGRP lines. Through classical genetic analyses, we found that it affects the immunodeficiency (Imd)-dependent enteric immune response through the induction of the major effector *Diptericin A* (*DiptA*). We also identified and in vivo validated a *cis*-regulatory variant in a predicted transcription factor (TF) binding site responsible for the difference in *ntc* expression between the resistance classes and validated the effect of the SNP on allele-specific gene expression in vivo. In this study, we thus leveraged the genetic tractability of the fruit fly, the ability to easily replicate experiments on the same genetic backgrounds, and investigation at the whole-organism level to characterize in depth the genetic and molecular mechanisms that contribute to gut immunocompetence variation in *Drosophila*.

## Results

### *Nutcracker* is the only gene that is significantly differentially expressed between resistance classes

**Fig. 1:**
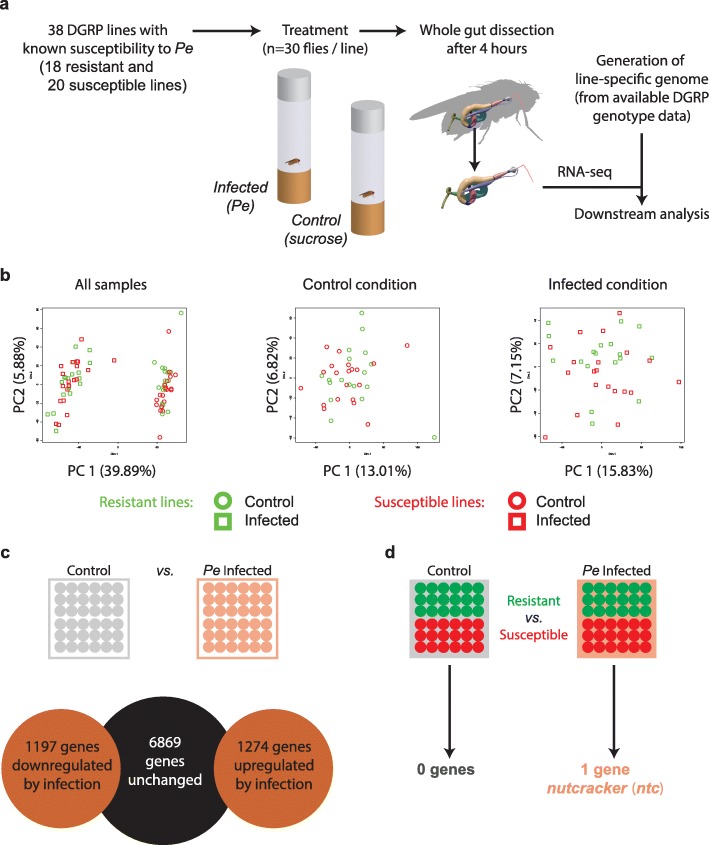
Expression profiling of phenotypic extremes does not reveal consistently differentially expressed genes between classes. **a** Study design: 30 adult female flies from two phenotypic extremes (18 resistant and 20 susceptible) of the DGRP were infected orally with *P.e.* or fed sucrose. Whole guts of ~ 30 flies were dissected per condition and line, then RNA sequencing was performed. Sequencing reads were mapped to individualized genomes, and the number of reads was counted per gene. **b** Principal component analysis plots of all the samples (left), the control condition (middle), and the infected condition alone (right). The R package FactomineR was used to obtain the coordinates of each sample in the first two components, as well as the variance explained by each component (in parentheses). **c** Infection leads to the differential expression of around 2400 genes (BH-corrected *p* value < 0.05, fold change > 2). **d** When lines of the two resistance classes are compared within condition, no genes are significantly differentially expressed in the control condition, and only one gene, *ntc*, in the infected condition

To study global gene expression variation between two enteric infection resistance classes, we selected 38 DGRP lines from the phenotypic extremes from our previous study [31] with 20 being highly susceptible and 18 being highly resistant to enteric infection by *P.e.* (Fig. 1a). Adult female flies were infected and mRNA sequencing (mRNA-seq) performed on dissected guts 4 h post infection. We chose this 4 h timepoint to detect acute gene expression differences and thus to avoid indirect expression changes that may occur because of gut remodeling. In parallel, for each line, we also sequenced guts of sucrose-fed flies as controls. Each genotype and condition were replicated once. Since the DGRP lines are highly polymorphic, we opted for analyses on individualized genomes. To do so, we used the available genotype data [33], including single nucleotide variants as well as indels and structural variations, to generate individualized genomes and gene annotations (see “Material and methods”) which we used throughout the analyses. Seven of the analyzed lines were already included in our previous study [31], which allowed us to assess the biological reproducibility of the mRNA-seq experiment. After combining the expression count data from the two experiments and performing normalization and removal of batch effects, we performed conventional hierarchical clustering (Additional file 1: Figure S1a). This revealed that the samples from the same line and condition always cluster together, indicating that genotypic differences mediate expression-level differences and that batch effects are weaker than the infection or genotype effects.

In a next step, we aimed to investigate how genetic variation influences the molecular and phenotypic differences between resistance classes. To first gain an unbiased, overall insight into the relatedness of the transcriptomes of the homozygous lines, we performed PCA on gene expression levels (Fig. 1b and Additional file 1: Figure S1b-c). While the infection effect is obvious and recapitulated by the first principal component (PC), lines from different resistance classes did not show any clear separation on the first two PCs. This is in contrast to our previous study, where we were able to see a modest separation on the second PC [31]. Furthermore, performing PCA on the expression levels within conditions yielded a similar result, with no obvious separation of the resistance classes on the first two principal components. A rationale for the disappearance of any separation compared to our previous study may include (i) our expansion of the number of lines (from 8 to 20 per extreme), therefore reducing the phenotypic spread, or (ii) the fact that the separation observed with the eight lines in our previous study may have been dominated by genotypic rather than treatment effects. Taken together, our findings suggest that, while the molecular impact of infection is similar among all tested lines and while the phenotypic differences are striking between the two resistance classes, the underlying transcriptomic differences are neither evident at the single gene- nor transcriptome-wide level. This is in line with our previous findings that higher-level modules related to specific biological processes such as stress response, ROS metabolism, and intestinal homeostasis [31] could explain differences between resistance classes.

Using standard gene-based differential expression analysis, we identified around 2400 genes that are either up- or downregulated 4 h post *P.e.* infection (FDR < 0.05, log fold change > 2, Fig. 1c). This is consistent with previous RNA sequencing and microarray results [31, 46]. Next, we explored gene expression differences between the resistance classes in the two experimental conditions. In our previous study, we had only found five and 34 mostly uncharacterized, differentially expressed genes in the control and infected conditions, respectively. We reasoned that this low number may reflect either the underpowered nature of our previous study, involving only four lines from each resistance class, or that there are effectively few consistent differences between the resistance classes at the single gene level. Strikingly, when considering 38 lines, we found again no differentially expressed genes in the control condition, and only one gene, *nutcracker* (*ntc*), in the infected condition (Fig. 1d). This observation supports the notion that the differences between the classes, while being overt at the physiological level (i.e., being alive vs. dead), cannot be fully explained at the single gene level using standard differential expression approaches, at least at the sampled 4 h post infection timepoint.

### The gene *nutcracker* is involved in the gut immune response

**Fig. 2:**
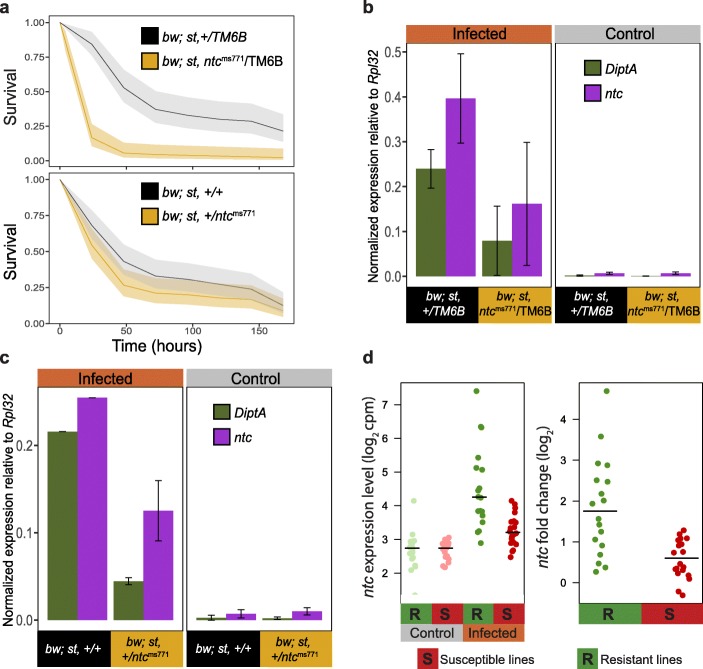
The gene *nutcracker* is involved in the gut response. **a** Survival of lines harboring a null mutant (yellow) allele *ntc*^*ms771*^ with (top panel) and without (bottom panel) TM6B balancer upon *P.e.* infection compared to control (black). Log ranked test *p* < 0.0001 and *p* = 0.081 for lines with and without balancer chromosome, respectively. Shaded area represents the 95% confidence interval. **b** Gene expression of *ntc* (purple) and *DiptA* (green) as measured by qPCR, normalized to *RpL32* in control (left) and infected (right) conditions in *ntc*^*ms771*^ mutant (yellow) and control (black) lines with TM6B balancer. **c** Gene expression of *ntc* and *DiptA* as measured by qPCR, normalized to *RpL32* in infected (right) and control (left) conditions in *ntc*^*ms771*^ mutant (yellow) and control (black) lines without TM6B balancer. Data presented in **a**–**c** are based on at least three biological replicates. **d** Left panel: Expression level (in log2(cpm)) of the *ntc* gene by resistance class in control (gray) and infected (orange) conditions. Right panel: Fold change of *ntc* expression by resistance class after infection. Green and red points represent resistant and susceptible DGRP lines, respectively

Because *ntc* had so far never been linked to the immune response, we first explored whether *ntc* affects gut immunocompetence given that its only described role is in sperm differentiation [47, 48]. To do so, we used a null mutant line that harbors a point mutation in the F-box domain of Ntc, *ntc*^*ms771*^ and tested its susceptibility to *P.e.* infection. Because flies homozygous for *ntc*^*ms771*^ are fragile and have a short lifespan in both control (Additional file 1: Figure S2a, log-rank test, *p* < 0.0001) and infected conditions (Additional file 1: Figure S2b, log-rank test, *p* < 0,0001), we backcrossed the fly line to itsbackground line (*bw;st*). We assessed the survival of F1 offspring compared to their control, i.e., we compared the survival of *bw;st,+/TM6B* to *bw;st,ntc*^*ms771*^*/TM6B* and *bw;st,+/+* to *bw;st,+/ntc*^*ms771*^. We observed decreased survival in all offspring flies harboring the *ntc* mutant allele. We also crossed the mutant line to *w*^*1118*^ and again scored survival, obtaining the same results. In both crosses, the decrease in survival was stronger in the balancer line compared to the one without a balancer chromosome (Fig. 2a, *p* < 0.0001 with balancer and *p* = 0.081 without balancer, log-rank test, Additional file 1: Figure S2d and S2e, cross with w^1118^, log-rank test *p* < 0.0001 with balancer, *p* = 0.9 without balancer). Furthermore, we performed RT-qPCR on dissected guts from the lines crossed to *bw;st* and found that *ntc* expression is, as expected, strongly reduced in mutant allele lines compared to control. Concurrently, the expression of the anti-microbial peptide *DiptA* was greatly reduced in flies harboring the *ntc* mutant allele compared to controls (Fig. 2b, c). We replicated these findings using two lines harboring P-element-induced mutations, *ntc*^*f03797*^ and *ntc*^*f07259*^, in or around the *ntc* locus, showing a decrease in survivability (Additional file 1: Figure S2c) and *DiptA* expression (Additional file 1: Figure S2f). Interestingly, we also found that *ntc* is not expressed in the *Rel*^*E20*^ mutant line, which harbors a Relish loss of function which disrupts the Imd pathway, upon infection (Additional file 1: Figure S2f). Furthermore, we observed that *ntc* expression is induced in the gut after infection (infection log_2_ fold change = 1.8, Benjamini-Hochberg adjusted *p* value = 7.87e^− 11^) and resistant lines have greater *ntc* expression than susceptible ones (log_2_ fold difference = 1.26, Benjamini-Hochberg adjusted *p* value = 0.009) after infection, but not in the control condition (Fig. 2d). Together, these results show that loss of *ntc* leads to enhanced susceptibility to *P.e.* infection and suggest that loss of or decreased *ntc* expression negatively influences the enteric immune response through downregulation of Imd pathway effectors upon *P.e.* infection.

### Genetic analysis reveals pervasive, condition-specific gene expression variation

**Fig. 3:**
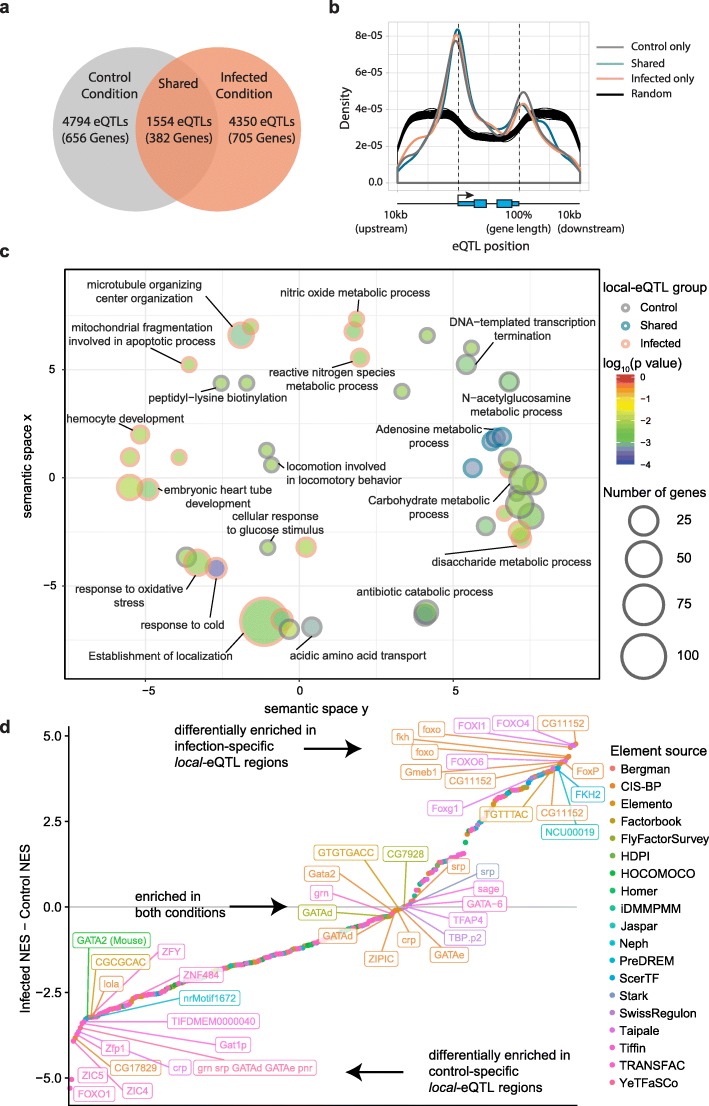
*local*-eQTL analysis links natural variation to gene expression levels. **a** Infection leads to the differential expression of around 2400 genes (BH-corrected *p* value < 0.05, fold change > 2). **b** Metaplot of the location of *local*-eQTLs with respect to their associated genes’ transcription start sites (TSS). The gray, blue, and orange lines represent the control, shared, and infected conditions respectively. Since SNP density is not uniform in the genic windows, the black lines represent the density of random samples of SNPs drawn from the pool of all SNPs that were included in the *local-*eQTL scan. Specifically, they are 100 samples of 5904 SNPs, which is equal to the number of detected *local-*eQTLs in the infected condition. **c** Graphical representation of enriched biological process gene ontology terms based on the lists of genes with significant *cis*-eQTL associations. The GO analysis was performed using the GOstats [50] R package (hypergeometric test *p* value < 0.005), and REVIGO [51] was used to reduce redundancy in the ontology groups and plot them by semantic similarity (allowed similarity = 0.7). The size of the circle indicates the number of genes belonging to a certain GO category, and the color indicates enrichment significance. The log_10_(*p* value) is the hypergeometric test FDR-corrected *p* value enrichment result. The *X*/*Y* space is the multidimensional scaling (MDS) of the pairwise semantic similarity measures (simRel). Closer terms in this 2D space imply closer GO terms (based on their similarity). **d** Differential enrichment of TF motifs around condition-specific *local*-eQTLs. Separate analyses were performed on i-cisTarget using regions of 201 bp centered around *local*-eQTLs specific to the control or infected condition. The difference between the infected and control normalized enrichment scores (NES) from the two analyses was calculated for each tested motif/feature. The color of the motif indicates the source database [52, 53]. The top 15 motifs from each side as well as the 15 motifs closest to 0 are labeled

We next sought to uncover the molecular mechanisms underlying differential *ntc* expression between resistant and susceptible lines by cataloguing the effect of genetic variation on gene expression levels including *ntc* for the two treatment conditions. To do so, we used Matrix eQTL [49] to identify *local-*expression Quantitative Trait Loci (*local*-eQTLs) (i.e., within a window of 10 kb up- and downstream of genes) whose alleles correlate with the expression levels of nearby genes. To avoid artificial inflation in the *p* values due to the correlation between two samples of the same strain derived from the control and infected conditions, we performed the analysis separately for the two experimental settings, while considering co-variates such as genetic relatedness and *Wolbachia* infection status (“Material and methods”). Using this model, we identified 6348 and 5904 *local*-eQTLs (Benjamini-Hochberg adjusted *p* value < 0.05 corresponding to a raw *p* value of 1.6e−4 and 1.4e−4, respectively) for 1038 and 1087 genes in the control and infected conditions respectively (Fig. 3a).

Interestingly, while 22% of *local*-eQTL-associated genes were shared between the two treatment conditions, the majority of detected *local*-eQTLs were condition-specific, emphasizing the substantial contribution of cryptic genetic variants to gene expression variation, especially in the presence of a strong transcriptome-altering stimulus such as infection. However, since the analyses were performed on only 38 strains, it is possible that statistical power limitations may be inflating the number of condition-specific *local*-eQTLs. To address this possibility, we first characterized the allele frequency spectrum of all significant *local-*eQTLs and found no systematic bias in allele frequency with respect to the number of identified *local-*eQTLs (Additional file 1: Figure S3a). For each *local-*eQTL, we then calculated the percentage of variance explained by genotype, and again found no clear relationship between allele frequency and the number of shared *local-*eQTLs (Additional file 1: Figure S3b). Subsequently, we performed simulations under idealized conditions for a wide range of allele frequencies and genetic contributions to variance, following a strategy described in [54] (Additional file 1: Figure S3c). These analyses revealed that the power to detect a *local-*eQTL has a broad range, implying that many condition-specific and shared *local-*eQTLs are likely not detected in our study at the lower bounds of the allele spectrum or genetic contribution. For example, the power to detect a *local-*eQTL with a MAF of 0.15 and a genetic contribution of 30% is 10% whereas it reaches 100% when the MAF is 0.5 and genetic contribution is 50%. We also simulated the power of detecting a shared *local-*eQTL by performing simulations in pairs that share the same genetic components but with a random environmental component. As expected, the power to identify a shared *local-*eQTL increases sharply as a function of increasing allele frequency and genetic contribution to trait variance (Additional file 1: Figure S3c). These simulations imply that if limited power leads to more condition-specific *local-*eQTLs, we should observe a relatively greater number of condition-specific *local-*eQTLs in the lower allele-frequency spectrum. However, we did not observe such a trend in our data. In fact, the odds of identifying condition-specific *local-*eQTLs versus shared *local-*eQTLs did not change as a function of allele frequency (Additional file 1: Figure S3d-f). Given these observations, we conclude that limited power cannot be a major reason for the observed, low number of shared *local-*eQTLs. Furthermore, we found that the meta-distribution of detected *local*-eQTLs around the respective transcription start sites (TSSs) is similar between the two conditions. The distribution also followed the expected pattern in that their density was highest around the TSS with a peak immediately downstream of the TSS, also involving the most significant associations (Fig. 3b). By defining genes that are expressed in the gut as genes with at least five reads in at least 38 samples out of 76, we further revealed that 26% of them could also be linked to at least one *local-*eQTL, reflecting pervasive genomic variation-mediated gene expression differences. Of particular interest is that we found 2 and 13 *local-*eQTLs linked to *ntc* in control and infected conditions respectively.

Because variation in the expression of *ntc* is unlikely to explain all by itself the difference in susceptibility to infection, we decided to use the generated *local-*eQTL dataset to uncover pathways affected by genetic variation. To do so, we performed Gene Ontology analyses on the control, infected, and shared set of *local-*eQTL genes. This analysis revealed few enriched terms in shared *local*-eQTL-associated genes. Genes linked to control-specific, *local*-eQTLs tended to be in metabolic processes, while infection-specific terms included terms related to response to oxidative stress, cold, reactive nitrogen species metabolism, and mitochondrial fragmentation (Fig. 3c). This suggests that genetic regulatory variation in the infected condition might be affecting distinct biological processes. To provide an additional layer of characterization, we explored whether infection-specific *local*-eQTLs are preferentially located in the proximity of *cis*-regulatory features / TF motifs. We considered a region of 200 bp around each eQTL and used i-cistarget [52, 53] to test for TF motif enrichment in infection- compared to control-specific regions. We found that regions from both tested conditions feature a similar enrichment of GATA TF motifs. Given the well-established role of GATA factors in gut development and homeostasis [55, 56], this result serves as a sanity check for our approach. Interestingly, regions surrounding infection-specific *local-*eQTLs were differentially enriched for motifs from the Forkhead box (FOX) TF family (Fig. 3d). Given that FoxO signaling is activated after oral bacterial infection and has been shown to be required for survival, we speculate that infection-specific *local*-eQTLs may be exposed by FoxO activation [57]. Taken together, our analyses catalogued a large set of genomic loci that affect gene expression levels only in the infected condition, collectively rendering them interesting candidates for a role in influencing the overall susceptibility of *Drosophila* to infection.

### Large-scale in vivo *local-*eQTL characterization via allele-specific expression

We have so far uncovered many shared and condition-specific *local-*eQTLs, but our analyses did not inform whether these *local-*eQTLs are *cis-* or *trans-*acting. For example, while we identified 13 *local-*eQTLs linked to *ntc* in the infected condition, we are at this point unable to characterize their precise mode of action, preventing insights into the underlying regulatory mechanisms. To validate the effect of a particular variant on relevant genes, eQTL studies have so far often resorted to classical molecular biology techniques such as chromatin immunoprecipitation and small-scale reporter assays [58, 59]. While the recent emergence of Massively Parallel Reporter Assays allows for a much more systematic analysis of the regulatory effect of variants in transcriptional elements [60–62], these assays are still unable to consider the complex interaction between genetic variation and gene expression.

**Fig. 4:**
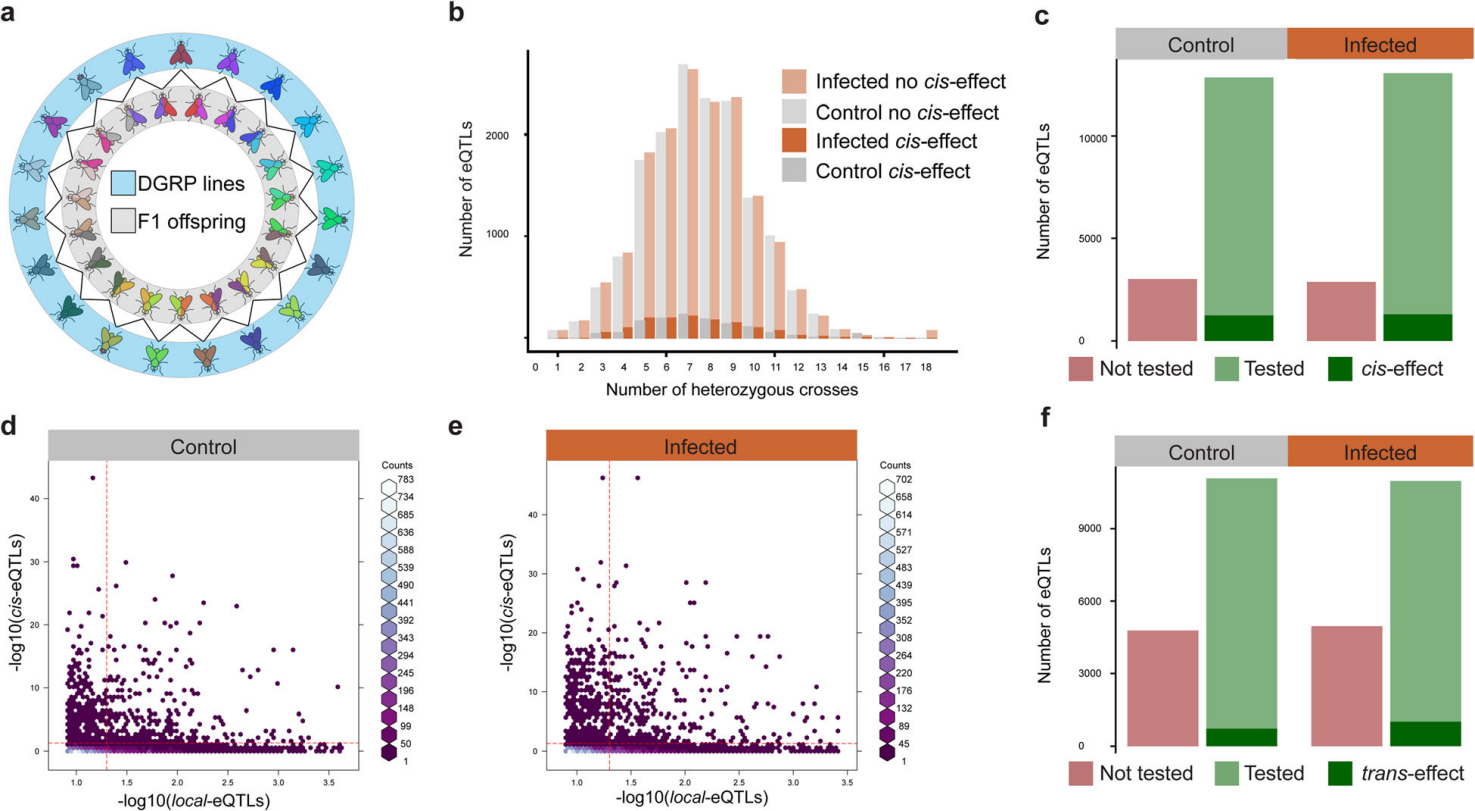
*local-*eQTL characterization by allele-specific expression reveals few *cis-*acting variants and a comparable distribution between *cis-* and non *cis-*eQTLs. **a** Schematic of the round robin design: isogenic parental lines (blue) were crossed to two different lines and heterozygote F1 female offspring (gray) were used for infection and further processing. **b** Distribution of the number of heterozygous crosses per eQTL in control (gray) and infected (orange) conditions. The distribution of *cis*-eQTLs (dark gray and dark orange) is not affected by the number of heterozygous crosses that are available to perform the calculations. **c** Number of eQTLs passing the data cutoff for *cis-*characterization (light green) and rejected (red) in control (19%) and infected (18%) conditions. *cis-*eQTLs are indicated in dark green with 9.7% and 7.5% of *local*-eQTLs in infected and control conditions respectively. **d**, **e** Correlation between *local-*eQTL *p* values (*x*-axis, −log10(Benjamini-Hochberg adjusted *p* value)) compared to *cis-*eQTL calculated *p* values (*y*-axis, −log10(Benjamini-Hochberg adjusted *p* value)). Vertical and horizontal lines represent the 0.05 cutoff in control and infected conditions. **f** Number of eQTLs passing the data cutoff for *trans-* characterization (light green) and rejected (red) in control (30.2%) and infected (31.2%) conditions. *Trans-*eQTLs are indicated in dark green with 6.6% and 9.3% of *local*-eQTLs in infected and control conditions, respectively

We therefore decided to exploit our experimental setting to thoroughly validate the detected *local-*eQTLs and explore their putative *cis-*regulatory nature by investigating their effect in a different genetic background*.* Specifically, by implementing a large-scale allele-specific expression analysis, we aimed at examining whether *local*-eQTLs induce the expected imbalance in expression between maternal and paternal alleles in an F1 cross [63, 64]. To achieve this, we selected 19 DGRP lines and crossed them in a round robin scheme (Fig. 4a and Additional file 1: Figure S4a) to maximize the number of F1 offspring that feature heterozygous genotypes for our set of predicted *local-*eQTLs, including those linked to *ntc*, such that we could assess allele-specific gene expression and infer *cis-*regulatory effects. Using the F1 individuals, we infected two to three- day-old adult females for 4 h and extracted RNA from their dissected guts. As a control, a similar number of female adults were fed sucrose and processed in similar fashion. We replicated this experiment to obtain two biological replicates and subsequently used BRB-seq, a high-throughput and cost-effective transcriptomics approach developed by our lab [65], to derive gene expression profiles for each of the processed samples (see “Material and methods”). Along with the F1 offspring, we also processed and sequenced four homozygous lines. We assessed the quality of the F1 offspring replicas after removing a sample for which downstream sequencing failed (Additional file 1: Figure S4b) by performing PCA and correlation analysis on the gene count matrix. The latter analysis revealed no major batch effects between replicate experiments and strong separation between infected and control samples (Additional file 1: Figure S4c-S4d). To benchmark our transcriptomic approach, we compared the four lines sequenced by both TruSeq and BRB-seq. We found that the two methods highly correlate on the number of counts for each gene in control (Additional file 1: Figure S5a-S5d, Pearson *r* > 0.82) and infected conditions (Additional file 1: Figure S5e-S5 h, Pearson *r* > 0.82) and on the fold change of differentially expressed genes (Additional file 1: Figure S5i, Pearson *r* = 0.795), consistent with previous results [65]. Together, these analyses demonstrate that our BRB-seq approach is able to recapitulate the original dataset at a raw level with similar read counts and information level with matching fold change.

We selected a subset of 15,851 eQTLs from the control condition and 15,923 ones from the infected condition among our detected *local*-eQTLs, with an FDR < 0.1 for further validation. To detect differential allele expression as driven by an eQTL-linked variant, we required at least one cross whose offspring would be heterozygous for the selected variant to assess allelic expression imbalance. To identify those crosses, we used the DGRP freeze 2 genomic data resource [33]. Even though we used only 19 DGRP lines, our design allowed us to comprehensively interrogate the majority of detected *local-*eQTLs on the subset of 38 lines. We thereby note that for an eQTL to be “testable,” the focal SNP must have lines that are heterozygous at this locus. Plotting the distribution of the number of lines that are heterozygous for one locus revealed that the average number of heterozygous crosses per *local-*eQTL variant is 6.5 for both the control and infected conditions, with only 70 and 72 *local*-eQTLs from the control and infected conditions (0.45% of *local-*eQTLs in both conditions) being not testable due to the absence of any F1 that is heterozygous at these loci (Fig. 4b). Although one *local-*eQTL is linked to one gene, it is possible that one gene may be affected by multiple *local-*eQTLs. The distribution of the number of *local-*eQTLs linked to each gene revealed that most genes are linked to one or two variants, with a maximum of 115 *local-*eQTLs linked to one gene. Moreover, we did not detect any difference in the distribution of *local-*eQTLs per gene between control and infected condition-linked *local*-eQTLs (Additional file 1: Figure S4f), indicating that having multiple *local*-eQTLs linked to one gene is unlikely to significantly influence our results.

To detect *cis-*eQTL variant-driven allele-specific expression (ASE) over several different genetic backgrounds, we applied a generalized linear mixed model (GLMM) with the response modeled by a binomial test of maternal vs. paternal reads and crosses as random effect. The binomial test has been widely used to detect allelic imbalance [66–69] and by adding the genetic background as a random effect, we can detect consistent allelic imbalance over multiple crosses. Thus, variants validated by our model are able to drive allelic imbalance across several genetic backgrounds. We applied strict cutoff parameters to the samples that were passed to the GLMM which eliminated approximately 19% and 18% of the *local*-eQTLs from the control and infected conditions respectively because those variants did not have sufficient reads or samples to be considered in the analysis (Fig. 4c, red bars). At the end, our model allowed us to uncover 9.7% of the control (1250 *local*-eQTLs with FDR < 0.05) and 7.5% of the infected (1301 *local*-eQTLs with FDR < 0.05) condition-linked *local*-eQTLs across all tested genetic backgrounds as *cis*-acting eQTLs (Fig. 4c, dark green bar). We next assessed if an increased number of F1 hybrids would result in a higher probability for a *local*-eQTL to be validated, but found no evidence for this (Fig. 4b). Interestingly, when we compared the adjusted *p* values computed by Matrix-eQTL for the *local*-eQTLs to the adjusted *p* values from the F1 data, we observed no correlation (Pearson *r* = 0.04 for the control condition and *r* < 0.01 for the infected condition), indicating that a low *p* value for a *local-*eQTL is not necessarily a good predictor of an actual *cis* effect across mixed genetic backgrounds (Fig. 4d, e). Furthermore, we found no correlation between the computed effect size and the measured effect size in both control and infected conditions (Additional file 1: Figure S6a and S6d). However, we observed that when a *local-*eQTL is found to act in *cis*, there is a high probability that the effect size calculated by Matrix-eQTL (called beta) accurately predicts the direction of the measured effect (Additional file 1: Figure S6b – S6c and S6e – S6f). We subsequently tested for a difference in the meta-distribution around the TSS between *local-* and *cis-*eQTLs and found that both distributions were comparable, with a greater density of non *cis*-eQTLs upstream of the gene and a greater density of *cis-*eQTLs downstream of the gene (Additional file 1: Figure S6 g and S6 h).

We then tested if *local-*eQTLs that were not characterized as *cis* could have a measurable *trans-*effect instead. To do so, we applied a linear mixed model to the crosses that were homozygous for each variant, using the crosses as a random effect (see “Material and methods”). We were able to detect a *trans*-effect for 6.6% of control and 9.3% infected condition non-*cis local*-eQTLs (727 and 1019 *trans-*eQTLs with FDR < 0.05 in control and infected conditions, respectively) (Fig. 4f). However, due to the restricted number of available homozygous crosses, we could only test 69.8% and 68.8% of the non-*cis local*-eQTLs in control and infected conditions respectively, while also being relatively underpowered. In summary, we detected a large number of *local-*eQTLs across conditions, but the majority of those cannot be defined as *cis-*eQTLs in a mixed heterozygous background. Rather, we found that, even within a conservative and underpowered analytical framework for *trans-*effect analysis, already a non-negligible portion of these non-*cis local*-eQTLs feature a robust, measurable *trans-*effect*.*

### Determining the *cis*-regulatory mechanism underlying differential *ntc* expression among resistant and susceptible DGRP lines

**Fig. 5:**
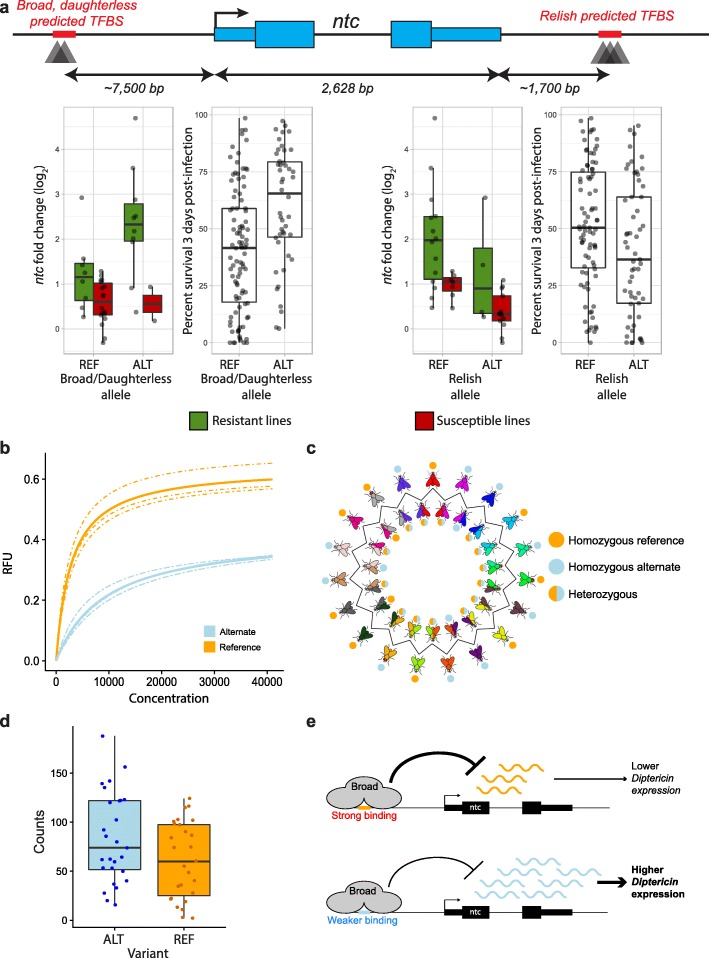
Broad binding affinity and *ntc* expression is lowered for the alternate allele. **a** Top panel: schematic of the *ntc* gene with specific annotations: *cis-*eQTLs around the *ntc* locus, and their overlap with predicted TF binding sites (TFBS). TFBS prediction was done using FIMO [70] and motifs from the Fly Factor Survey [71] and OnTheFly [72] databases. The expression fold change of *ntc* by resistance class and two of those alleles (termed the *broad*/*daughterless* allele (left panel), and the *relish* allele (right panel)) is plotted, as well as the survival percentage of 140 DGRP lines [31]. Green and red boxplots represent resistant and susceptible DGRP lines, respectively. **b** Measure of the binding affinity between Broad and the reference or alternate allele as measured by MITOMI [73, 74] in three different replicates. **c** Repartition of lines in the round robin scheme based on reference or alternate *broad* TFBS alleles. **d** Ratios of read count mapping to the alternate over the reference allele reveal no difference in control condition (*t*-test, *p* value = 0.21) but is significant in the infected condition (*t*-test *p* value = 0.04). **e** Proposed model of *ntc-*mediated variation in gut immunocompetence: an enteric immune challenge increases *ntc* expression, while Broad acts as a repressor of *ntc* expression. The SNP in the Broad binding site decreases the binding affinity for Broad and thus the extent of *ntc* repression, resulting in greater *ntc* expression, which in turn increases *DiptA* expression and overall gut immunocompetence

Next, we exploited the generated datasets to specifically elucidate the regulatory mechanisms underlying *ntc* expression variation given that it is the only differentially expressed gene between the resistant and susceptible lines (Fig. 1d), that it is linked to several *local-*eQTLs, and that resistant lines tend to have greater *ntc* expression than susceptible ones (Fig. 2d). Mining of our *local*-eQTL data revealed five infected condition-specific *local-*eQTLs belonging to two SNP clusters, one group consisting of two eQTLs 7.6 kb upstream and the other group composed of three 4.5 kb downstream of its TSS (Fig. 5a). These observations raised the question whether putative *cis-*regulatory variation of *ntc* expression could be one of the likely several mechanisms that contribute to resistance class stratification.

To test this postulate, we first performed a TF motif scanning analysis of the *ntc* locus. This revealed several potential TF binding sites (TFBS) that overlapped with the *ntc*-linked *local-*eQTL SNP clusters, including Broad Complex and Daughterless sites for upstream *local-*eQTLs, and a Relish/NF-kB one for a downstream *local-*eQTL. The alleles at both sites showed a high correlation with *ntc* expression for the studied 38 lines. But when associated with enteric infection susceptibility variation among the 140 DGRP lines, the allele at the Broad/Daughterless site was more significant than the Relish/NF-κB binding site one (Fig. 5a, GWAS *p* value of 6.1 × 10–5 vs. 0.024 respectively), even though both failed to pass the stringent, implemented nominal 1 × 10–5 *p* value [31]. In addition, since the gene *IntS10* is physically closer to these variants than *ntc*, we would not intuitively have linked these variants to *ntc*.

Because a SNP in a TFBS could disrupt binding of the respective TF [75], we next investigated the impact of the *local*-eQTL variant on the binding activity of the four different TFs predicted to bind the sites overlapping *ntc*-linked *local*-eQTLs: Broad, Daughterless, Sage, and Relish. To do so and given the difficulty in performing line-specific ChIP on these TFs, we used our in-house MITOMI setup [73] to measure in vitro the binding affinity of the selected TFs to double-stranded 20-mers that encompassed the respective binding site and that represented either the reference or alternate alleles. These analyses showed that among all four tested TFs, only Broad, a protein able to act both as a repressor and an activator [76, 77], exhibited a differential binding activity (Fig. 5b and Additional file 1: Figure S7, Welch’s *t*-test *p* value = 0.0063), showing substantially reduced binding to the alternate compared to the reference binding site allele.

Because the increase in *ntc* expression upon infection is substantially higher in DGRP lines harboring the alternate Broad binding site allele and because the alternate allele has a weaker affinity for Broad, we hypothesized that Broad, in our study, acts as a repressor on *ntc*. Consequently, a decrease in Broad binding affinity would lead to less repression and thus increased *ntc* expression. To verify this hypothesis in vivo, we again turned to the round robin F1 BRB-seq data to measure the ASE at each variant, with 14 F1 lines being heterozygous for the focal *ntc* variant (Fig. 5c). In our genome-wide ASE analysis, we used a stringent threshold defined as the minimum number of total reads superior to the maximum value between 6 or the 25th quantile of the total of reads assigned to the lineage lines in each sample (see “Material and methods”). We used this threshold to eliminate false positives due to low read mapping issues, in particular for genes that are lowly expressed. However, since the lowest number of reads mapping to *ntc* was 35, implementation of this stringent threshold was no longer required. Hence, we applied the same generalized mixed model used for our genome-wide ASE analysis on all *ntc* heterozygous samples, but without any threshold, consistent with comparable analyses in previous studies [69, 78, 79]. Using this analytical strategy, we found a significant effect of the variant on allelic imbalance for the infected condition, with higher counts to the alternate over the reference allele (Fig. 5d, *p* value = 0.042). These findings suggest that the variant in the Broad TF binding site is a *cis-*acting eQTL that affects *ntc* expression. Together, these results present a compelling mechanism explaining how a variant located in a TFBS contributes to variation in gut immunocompetence by altering the expression level of a particular gene that itself influences an organism’s resistance to infection.

## Discussion

This study aimed to elucidate the effect of genetic variation on gene expression and organismal phenotypes in the context of in vivo enteric infection in the DGRP. One of the major findings that emerged is that DGRP lines with diametrically opposite resistance to infection all have a similar response after ingestion of a pathogenic bacterium, at least at an early timepoint after infection (Fig. 1). We show that this is not due to our inability to detect genotype-specific differences, since lines of the same genotype cluster together at the transcriptional level (Additional file 1: Figure S1). It is therefore clear that genomic variation imparts line-specific systemic differences on the transcriptome, yet only a small subset of those differences appears to be relevant in determining resistance.

To directly assess the effect of genomic variation on gene expression levels, we catalogued the possible *local*-eQTLs around all expressed genes. We found that in both the control and infected conditions, around a third of all associations are unchanged, confirming that genotypic variation indeed drives gene expression differences. However, the majority of *local-*eQTLs proved to be condition-specific, including the *local-*eQTLs at the *ntc* locus*.* Since we demonstrate that limited power cannot adequately explain this observation, our data suggest that cryptic variation has an important contribution to infection resistance [80, 81]. Furthermore, our study allowed us to acquire unique insights into the regulatory nature of detected *local-*eQTLs. Most notable is that our study, to our knowledge the most comprehensive and systematic in vivo *local-*eQTL characterization effort to date, indicates that we tend to vastly overestimate the frequency of *cis-*eQTLs. This conclusion is in line with a previous study on mice in which only 17% of *local-*eQTLs could be defined as *cis-*eQTLs [44]. Moreover, while we were able to still classify many *local-*eQTLs as *trans*, the majority of *local-*eQTLs remained unvalidated in variable genetic backgrounds*.* Of course, it is possible that the *cis* effect of a *local*-eQTL may be masked by other *trans-*acting eQTLs affecting the same gene [5]. Indeed, when several eQTLs were predicted to affect one gene, we were not able to disentangle their effects. In addition, a single polymorphism may drive differential expression and the other eQTLs may be merely in linkage disequilibrium (LD) with the effector SNP. It is also possible that a given variant is able to affect a gene only in a small set of genetic backgrounds and thus even more crosses would be required to increase the number of testable heterozygous genomic sites. Several confounding factors may also influence these validation numbers, including the fact (i) that some variants may affect different target genes that are located farther away (e.g., in the case of intergenic variants) or that are even separated from the variant by other genes and (ii) that some variants only affect a gene in combination with other variants [82]. Importantly though, even if only considering the validated *cis-*eQTLs, our earlier statement of pervasive, condition-specific gene expression variation between genotypes remains intact, since 10% of the validated *cis-*eQTLs were condition-specific. Interestingly, we found that highly significant *local*-eQTLs were not necessarily more likely to act in *cis*. However, when a variant was characterized as a *cis-*eQTL, then the *local*-eQTL measured effect directionality was a good indicator of the *cis-*eQTL measured one.

Strikingly, we found only one gene that is differentially expressed between the resistant and susceptible lines, *nutcracker* (*ntc*). This gene was initially identified in a screen for mutants that failed to undergo sperm individualization due to their inability to activate caspases [47]. Through its F-box domain, Ntc interacts with other partners to form an SCF (Skp, Cullin, F-box) ubiquitin ligase (E3) complex that controls caspase activity in *Drosophila* [48]. Caspases play important roles in insect immunity and homeostasis through both apoptotic and non-apoptotic pathways. For instance, Dredd, the homolog of human Caspase-8, is required for Relish cleavage and activation [83]. Furthermore, activation of the IKK complex is dependent on ubiquitination [84], and studies in mammals have shown that commensal bacteria can affect ROS levels, leading to modification of the activity of the SCF complex, thus affecting NF-κB signaling [85]. While there are therefore several possible functional scenarios, the exact function of Ntc in the gut and specifically enteric infection remains unclear and should be the subject of a more mechanistic, follow-up study. However, we were able to demonstrate that impaired *ntc* expression and null mutants of *ntc* negatively impact the survival of flies harboring these mutations. Intriguingly, low *ntc* expression does not correlate with susceptibility in the DGRP lines. This could be interpreted as a result of the sum of several different factors that are, when taken individually, not impactful, but lead to an increase in susceptibility when combined, as is suggested by our RNA-seq results not displaying strong separation between resistant and susceptible lines. Moreover, we were able to show that *DiptA* expression is severely reduced in the absence of *ntc*, showing a direct impact of *ntc* expression on potent immune response effectors.

We thereby uncovered how a SNP in a TFBS proximal to *ntc* may impact its expression upon enteric infection. It is by now well-established that variants in TF binding sites can impact binding affinity and in turn the expression of the respective target gene [8, 86]. Here, we found that only one mutated binding site out of two possible *local-*eQTL sites displays variable binding affinity to a TF, namely Broad. Furthermore, allele-specific expression of F1 hybrids carrying the two alleles showed that the two copies of *ntc* are being induced differently, demonstrating a *cis* effect of the SNP on the expression of *ntc*. These results suggest a causal relationship between the binding site variant and variable *ntc* expression through potential differential binding of the TF Broad, constituting to our knowledge a rare example of an eQTL that modifies an ecologically relevant complex trait through its effect on binding of a specific TF in a particular environmental condition. That said, it is unlikely that the extreme phenotype observed for *ntc* mutants reflect all of the underlying molecular mechanisms differentiating the resistant and susceptible DGRP lines since the difference in *ntc* expression between susceptible and resistant lines is not as severe as those measured in the mutants.

Together, these observations support the following model regarding how the *ntc* locus mediates variation in enteric infection susceptibility (Fig. 5e): upon infection, the expression of *ntc* is increased, together with that of *broad* as well as several other immune response genes, as inferred from [46, 87]. Given Broad’s role as a repressor in metamorphosis [88], we hypothesize that this TF may also act as a negative (feedback) regulator of *ntc* expression. Consequently, in flies harboring the alternate allele showing diminished affinity for Broad binding, *ntc* repression is reduced, resulting in greater *ntc* expression. This in turn positively affects the expression of *DiptA* through an as yet unknown mechanism, resulting in greater infection resistance compared to susceptible lines.

## Conclusions

Our study shows the advantage of allele-specific experiments as a complement to standard eQTL approaches to identify causal variants as well as the power of systems genetics to assign novel roles to genes in biological processes unrelated to their originally discovered roles. During our research, we did not consider the fact that the gut is a highly regionalized organ [89, 90] that consists of multiple cell types [91]. It is possible that some eQTLs could therefore be restricted to a certain cell type or environment, which cannot be detected using our current strategy, but could be investigated in a follow-up study.

## Material and methods

### Fly stocks

DGRP lines were obtained from the Bloomington stock center and reared at room temperature on a standard fly medium with 12-h light dark cycle. The fly medium we used is composed of (for 1 L water): 6.2 g Agar powder (ACROS N. 400,400,050), 58.8 g Farigel wheat (Westhove N. FMZH1), 58.8 g yeast (Springaline BA10), 100 ml grape juice, 4.9 ml Propionic acid (Sigma N. P1386), 26.5 ml of methyl 4-hydroxybenzoate (VWR N. ALFAA14289.0) solution (400 g/l) in 95% ethanol. We used *w*^*1118*^ and *bw;st* flies as wildtype. Various DGRP lines, *ntc*^*f03797*^ and *ntc*^*f07259*^ stocks were obtained from the Bloomington Stock Center. The *bw;st,ntc*^*ms771*^*/TM6B* mutant stock was a kind gift from the Hermann Steller lab.

### Oral infection

Oral infection was performed as previously described [92]. Briefly, 1-day-old females were transferred to 29 °C rearing conditions. When the female flies were 2–3 days old, they were starved for 2 h and then transferred to a tube containing bacteria and allowed to feed on the bacteria for a maximum of 24 h. To prepare the *P.e.* bacterial pellet, bacteria were plated from glycerol stocks on a standard LB-agar plate supplemented with 1% milk and grown overnight at room temperature. Two days prior to infection, one single colony was transferred to a 50-ml Erlenmeyer with 12.5 ml LB and incubated for 8 h at 29 °C with 180 rpm shaking. The pre-culture was then transferred to a 1-L Erlenmeyer with 200 ml LB and the culture was incubated overnight using the same conditions as the pre-culture. The culture was then centrifuged at 2500*g* at 4 °C for 20 min. The remaining LB was discarded, and the pellet was resuspended by pipetting up and down. The OD600 was measured using a CO8000 Cell density meter. The pellet was then diluted to a final OD600 of 100 with distilled water and supplemented with Sucrose to a final volume/volume of 1.25%. A control solution contained only Sucrose at the same concentration. A disc of Whatman paper was layered on top of the food and 225 μl of the bacterial or control solution was added to the paper.

### Survival

Flies were infected as described previously. Four hours after infection, surviving flies were scored. After 24 h of feeding on bacteria, flies were transferred to fresh tubes and survivors were scored. Then, every 24 h, survivors were scored and flies were transferred to fresh tubes every 48 h. The R package Survival was used to compute the log-rank test to assess statistical differences between genotypes. The analysis was performed in R 3.5.1.

### qPCR

RNA was extracted using the same method as for the BRB-seq library preparation described above. cDNA was synthesized from 500 ng total RNA using *SuperScript II* enzyme (Thermo Fisher 18064014). qPCR experiments were performed on a StepOnePlus Real-Time PCR system (Applied Biosystems) using the Power SYBR® Green PCR Master Mix (Applied Biosystems). Gene expression relative to the housekeeping gene *RpL32* was calculated separately for each biological replica.

List of primers used:

**Table:** 

*ntc* Forward	GATCAGGTGGGGAAAAAGCAG
*ntc* Reverse	GTTGTTCGCTCAGGATTCGC
*DiptA* Forward	GCTGCGCAATCGCTTCTACT
*DiptA* Reverse	TGGTGGAGTGGGCTTCATG
*RpL32* Forward	GACGCTTCAAGGGACAGTATCTG
*RpL32* Reverse	AAACGCGGTTCTGCATGAG

### RNA sequencing on DGRP lines, differential gene expression, and *local*-eQTL analysis

#### RNA extraction

Guts from 30 adult female flies were freshly dissected in PBS after 4 h of infection with a pellet of *Pseudomonas entomophila* at OD100. The guts were then transferred to 1000 μl Trizol Reagent (Invitrogen) with 10 μl plastic beads, then homogenized in a Precellys 24 Tissue Homogenizer at 6000 rpm for 30 s. RNA extraction was performed using the manufacturer’s protocol. The RNA pellet was resuspended in 8 μl of RNAse-free water prior to Nanodrop quantification and quality verification, followed by final dilution to a concentration of 500 ng/μl.

#### Library preparation and sequencing

Standard Illumina Truseq libraries were prepared from 1 μg total RNA as measured by a Nanodrop 1000 device (Thermo Scientific) by the Lausanne Genomic Technologies Facility. Single end sequencing was performed for 100 cycles. Initially, 80 samples from 40 lines were sequenced but we excluded 4 samples from two lines. One of the lines was contaminated, as its reads were derived from two genotypes and another DGRP line had a smaller library size in one condition, with led to its elimination from the analysis.

#### Mapping to individualized genomes

To avoid bias in estimating gene expression levels due to known genetic variation, we generated an individualized fasta genome sequence for each DGRP strain based on homozygous variants in the published Freeze 2 DGRP genotypes and the Release 5 reference genome. We chose homozygous variants since any variants called as heterozygous at the time of DNA sequencing may either have remained heterozygous or may have become fixed in our stocks. Any heterozygous locus was assumed to carry the reference allele. We also generated individualized gene annotations by applying the offsetGTF tool included in the mmseq package [93] on the Ensembl BDGP5.25. For each sample, reads were mapped to the respective genome using STAR aligner. Reads for each gene were counted using HTseq-count.

#### Normalization and differential expression

We used the edgeR package to perform TMM normalization, followed by conversion to Counts Per Million using Voom with quantile normalization. When we combined samples from this study and the previous study, we used the same approach, starting from combined gene counts, with the addition of the removeBatchEffect function in the limma package. Differential expression was performed in limma using the weights obtained by Voom while adjusting for intra-line correlations using the duplicate correlation function with the DGRP lines as the blocking factor. The following model was used: *y* = treatment + class + treatment:class with “treatment” being the infected status and “class” the resistant or susceptible status. For each predictor variable, genes having a fold change of 2 and a Benjamini-Hochberg corrected adjusted *p* value of 0.05 were deemed differentially expressed.

#### Principal component analyses

The FactoMineR package was used to perform the principal component analyses on log2 count per million data as normalized by Voom after keeping expressed genes (count > 5 in more than 38 samples). PCA was performed with scaling and centering to avoid biases from differences in gene average expression or length.

#### *local-eQTL* analysis

We performed separate analyses for each infected condition with Matrix-eQTL using a linear model that accounts for genetic relatedness and *Wolbachia* infection status [49]. Variants that are within 10 kb of an expressed gene and whose minor allele frequency (MAF) is greater than 5 in the 38 tested lines were kept in the analysis. MAF here is actually the number of lines carrying the less prevalent allele in the sampled strains divided by 38. This translates to a minimum of 6/38 = 15.8%. To account for genetic relatedness, we calculated the three genotype principal components using the SNPrelate R package using a pruned set of SNPs from the DGRP freeze 2 genotypes (ld threshold = 0.2). *Wolbachia* infection status was obtained from the DGRP2 resource website (http://dgrp2.gnets.ncsu.edu/). Associations with a *p* value less than 0.001 were kept, followed by FDR estimation using the Benjamini-Hochberg procedure as implemented in Matrix-eQTL. Each gene’s expression level was transformed to a standard normal distribution based on rank. *Local*-eQTL associations with an FDR-corrected *p* value lower than 0.05 were considered significant. Metaplots were plotted in R. The GO analysis was performed using the GOstats [50] R package (hypergeometric test *p* value < 0.005), and REVIGO [51] was used to reduce redundancy in the ontology groups and plot them by semantic similarity (allowed similarity = 0.7). For each pair of significant GO terms, Revigo calculates Resnik’s and Lin’s semantic similarity (simRel) [94]. The two-dimensional representation is the result of multidimensional scaling (MDS) applied to the terms’ semantic similarity matrix.

#### TF motif enrichment

To determine TF motif (regulatory feature) enrichment in regions around condition-specific eQTLs, we generated a BED file of the genomic coordinates of a window of 201 bases centered around each *local*-eQTL. We then submitted this file to i-cisTarget [52, 53] with the following settings: analysis type = Full analysis; Species = *Drosophila melanogaster* (dm3); database version 5.0, and all features selected. After performing the two analyses, we used the comparison tool on the website to determine differential TF motif enrichment between the infected and control-specific *local*-eQTL genomic regions.

All analyses were performed in R version 3.5.0.

### Round Robin BRB-seq and allele-specific expression analysis

#### RNA extraction

Flies were killed in cold 70% ethanol, the ethanol was wiped and replaced with cold RNAse-free 1× PBS supplemented with 0.02% Tween-20. Ten guts were dissected for each sample and placed in a screw cap Eppendorf tube containing 350 μl Trizol and 10 μl plastic beads. Samples were homogenized in a Precellys 24 Tissue Homogenizer at 6000 rpm for 30 s. Samples were then transferred to liquid nitrogen for flash freezing and stored at − 80 °C. For RNA extraction, tubes were thawed on ice, supplemented with 350 μl of 100% ethanol before homogenizing again with the same parameters. We then used the Direct-zol™ RNA Miniprep R2056 Kit, with the following modifications: we did not perform DNAse I treatment, we added another 2 min centrifugation into an empty column after the RNA wash step, finally elution was performed by adding 10 μl of RNAse-free water to the column, incubation at room temperature for 2 min, and then centrifugation for 2 min. RNA was transferred to a low-binding 96-well plate and stored at − 80 °C.

#### BRB-seq library preparation

RNA quantity was assessed using picogreen. Samples were then diluted to an equal concentration in 96-well plates. RNA was then used for gene expression profiling using the bulk RNA barcoding and sequencing (BRB-seq) approach recently developed by our lab [65]. This protocol is able to provide high-quality 3′ transcriptomic data by implementing an early multiplexing scheme as in single-cell protocols and at a fraction of the cost of its competitors (e.g., 10-fold lower than Illumina Truseq Stranded mRNA-seq). In short, the BRB-seq protocol starts with oligo-dT barcoding, without TSO for the first-strand synthesis (reverse transcription), performed on each sample separately. Then all samples are pooled together after which the second-strand is synthesized using DNA PolII Nick translation. The sequencing library is then prepared using cDNA tagmented by an in-house produced Tn5 transposase preloaded with the same adapters (Tn5-B/B) and further enriched by limited-cycle PCR with Illumina compatible adapters. Libraries are then size-selected (200–1000 bp), profiled using a High Sensitivity NGS Fragment Analysis Kit (Advanced Analytical, #DNF-474), and measured using a Qubit dsDNA HS Assay Kit (Invitrogen, #Q32851). Finally, 6–8 pg of libraries was sequenced twice with Illumina NextSeq 500 with 21 cycles for read 1 (R1) and 101 cycles for read 2 (R2), only for the second sequencing.

#### Alignment

We first aligned the two libraries, only the R2 file, to the *Drosophila* reference genome release 3 and the BDGP5.25 release annotation using STAR 2.5.3a [95] with the following relevant parameters: --twopassMode Basic --outFilterMultimapNmax 1 --outSAMmapqUnique 60. Then we used an in-house built software (https://github.com/DeplanckeLab/BRB-seqTools) to annotate the two aligned BAM files with the R1 info (Barcode and UMI if the latter exists), generating read groups for each libraryXsample. Then the two BAM files were merged into a unique BAM file that was further sorted. Picard was then used to remove the duplicates using the read group information and the barcode tag (options BARCODE_TAG = BC READ_ONE_BARCODE_TAG = BX). One of the samples failed due to a very low amount of reads and was removed from further analysis (Additional file 1: Figure S2b). We then used PicardTools (http://broadinstitute.github.io/picard) to add read groups, sort, index, and remove duplicates using the UMI information (parameter BARCODE_TAG = BC READ_ONE_BARCODE_TAG = BX). We then used GATK [96] to split N cigars reads and realign the reads following the GATK best practices [96]. Finally, we used an in-house built software that assigns the reads to the maternal or paternal lines based on the variants present in the read, using the DGRP Freeze 2.0 VCF file [33].

#### Allelic imbalance measurement

For each *local*-eQTL and its linked gene, we used the variant information from the vcf file to select only crosses that were heterozygous for the respective variant. Using the same file, we further characterized each parental or maternal line as alternate or reference for each SNP. We then constructed a matrix with the raw number of reads mapping to the gene linked to the eQTL and classify them as either reference or alternate. We then applied a generalized linear mixed model (GLMM, R package lme4::glmer, binomial (alternate read count, reference read count) ~ (1|cross)) with the response modeled by a binomial distribution based on the number of reads mapping to each parental line with the crosses as random effects and no fixed effect. For each *local-*eQTL, we only selected samples with a minimum number of reads superior to the maximum value between 6 or the 25th quantile of the total of reads assigned to the lineage lines in each sample. The obtained *p* values were then adjusted using the Benjamini-Hochberg method. The effect size was computed as the inverse logit of the estimated intercept computed by the GLMM function.

#### *Trans*-effect measurement

For each *local*-eQTL and its linked gene, we used the variant information to select only crosses that were homozygous for the variant. We used the log2 count per million of total read count normalized using Voom after correction for batch effect and assigned them as alternate or reference variant. We then applied a linear mixed model (GLMM, R package lme4::lmer, log2(cpm) ~ variant + (1|cross)) using the normalized count as a response and modeled by the allele (reference or alternate) and the crosses as random effects. For each *local*-eQTL, we only selected samples with at least two homozygous crosses for each variant. The obtained *p* values were then adjusted using the Benjamini-Hochberg method.

All analyses were performed in R version 3.5.1.

#### Comparison between TruSeq and BRB-seq data

We selected only the homozygous lines that were sequenced along with the F1 offspring. We followed the same steps as the ones performed on the TruSeq samples, namely we used the edgeR package to perform TMM normalization, followed by conversion to counts per million using Voom with quantile normalization. We then used the removeBatchEffect function from the limma package. Differential expression was performed in limma using the weights obtained by Voom while adjusting for intra-line correlations using the duplicate correlation function with the DGRP lines as the blocking factor. The following model was used: *y* = treatment + genotype.

### MITOMI

All target DNA fragments were obtained as single-strand oligonucleotides from IDT. These oligonucleotides were subsequently used to generate labeled double-stranded oligonucleotides as described previously [73]. TFs were expressed in vitro using the TnT SP6 High-Yield Wheat Germ protein expression system (Promega) with a C-terminal eGFP tag. The surface chemistry, MITOMI, and image acquisition were performed as described previously [73, 74]. We quantified the amount of each mutated sequence that is bound to the respective TF at the equilibrium state by means of fluorescence in a range of six input DNA concentrations. The obtained kinetic binding curves for each sequence were then fitted with the non-linear regression function according to the Michaelis-Menten law.

## Supplementary information

**Additional file 1: Figure S1.** Reproducibility of line-specific transcriptomes. **Figure S2.** Analysis of several *ntc* mutants. **Figure S3.** Relationship between minor allele frequency and condition-specific local-eQTLs. **Figure S4.** Quality control of BRB-seq libraries. **Figure S5.** Comparison between TruSeq and BRB-seq libraries. **Figure S6.** Comparison of measured *local-*eQTL effect size and *cis-*eQTL effect size. **Figure S7.** MITOMI analysis of distinct TFs associated with the *ntc* locus.

**Additional file 2.** Review history.

## Data Availability

RNA-seq and Round Robin BRB-seq libraries have been deposited to the GEO repository (RNA-seq: GEO GSE118142 and BRB-seq GEO GSE138801 [97, 98]). All other data generated or analyzed during this study are included in this published article [and its Additional file information files] and in the laboratory github (https://github.com/DeplanckeLab/Frochaux_BouSleiman_2020) [99].
