# Supplementary material for: cis-regulatory variation modulates susceptibility to enteric infection in the Drosophila genetic reference panel
Source: Genome Biol. 2020 Jan 17;21:6. doi: 10.1186/s13059-019-1912-z (PMC6966807; doi:10.1186/s13059-019-1912-z)
Supplement: Supplementary file 1 — Additional file 1: Figure S1. Reproducibility of line-specific transcriptomes. Figure S2. Analysis of several ntc mutants. Figure S3. Relationship between minor allele frequency and condition-specific local-eQTLs. Figure S4. Quality control of BRB-seq libraries. Figure S5. Comparison between TruSeq and BRB-seq libraries. Figure S6. Comparison of measured local-eQTL effect size and cis-eQTL effect size. Figure S7. MITOMI analysis of distinct TFs associated with the ntc locus. [file 13059_2019_1912_MOESM1_ESM.docx]

**Additional file 1**


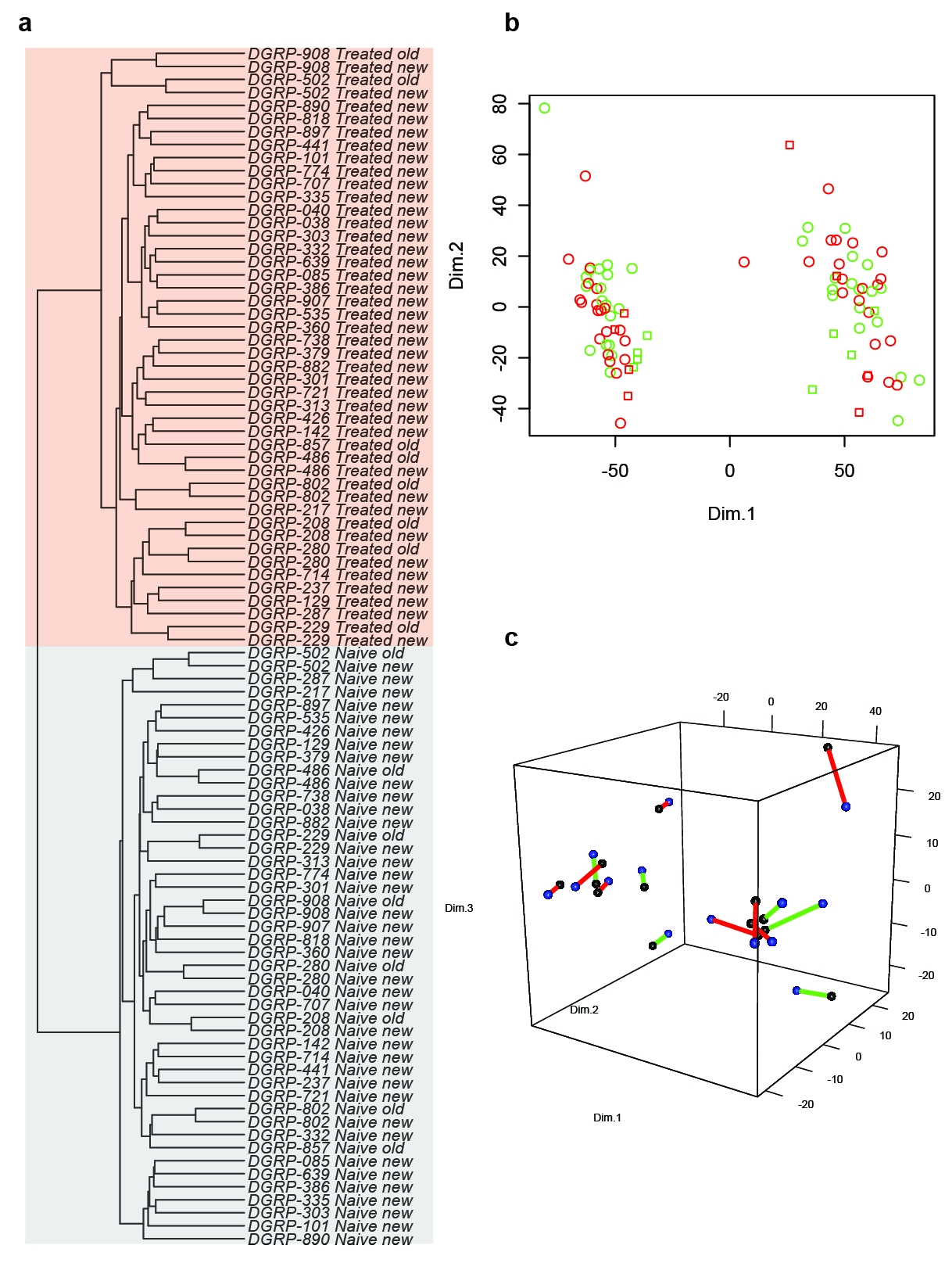


# Fig S1: Reproducibility of line-specific transcriptomes

1. Hierarchical clustering of the combined samples from this study and the previous one (1). Hclust was used on the Euclidean distance matrix in R.
2. Principal component analysis based on the gene expression profiles of the combined samples. Samples from the new and old study are represented as circles and squares, respectively.
3. Three-dimensional representation of the first three principal components based only on the samples that belong to lines replicated between the two studies. Corresponding samples are connected by a segment that is colored based on susceptibility group. The sphere color indicates the batch (blue is new, black is old) with green being resistant and red being susceptible lines.


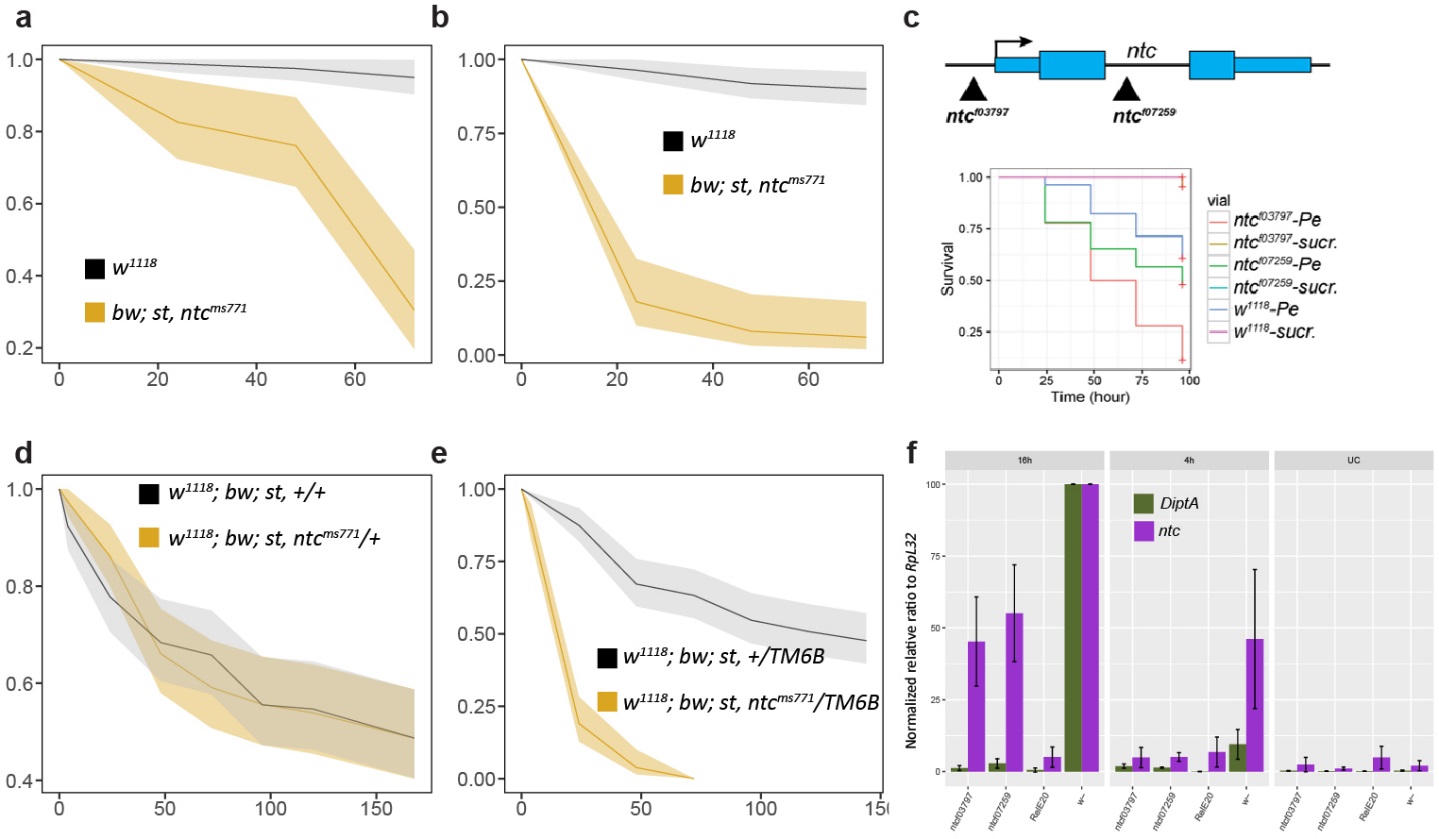


# Fig S2: Analysis of several *ntc* mutants

1. Survival of *bw; st, ntc^ms771^* homozygous flies in the control condition.
2. Survival of *bw; st, ntc^ms771^* homozygous flies in the infected condition.
3. Top panel: location of p-element insertions; bottom panel: survival of P-element lines upon *P.e.* infection.
4. Survival of *bw; st, ntc^ms771^* mutants crossed with *w^1118^* with TM6B balancer chromosome.
5. Survival of *bw; st, ntc^ms771^* mutants crossed with *w^1118^* without TM6B balancer chromosome.
6. qPCR-based expression of *ntc* and *DiptA* normalized to *RpL32* in P-element lines, *w^1118^* and *Rel^E20^* in control and infected conditions. Data from at least three biological replicates.

Data presented in (a), (b), (c), (d), (e) and (f) are based on at least three biological replicates.


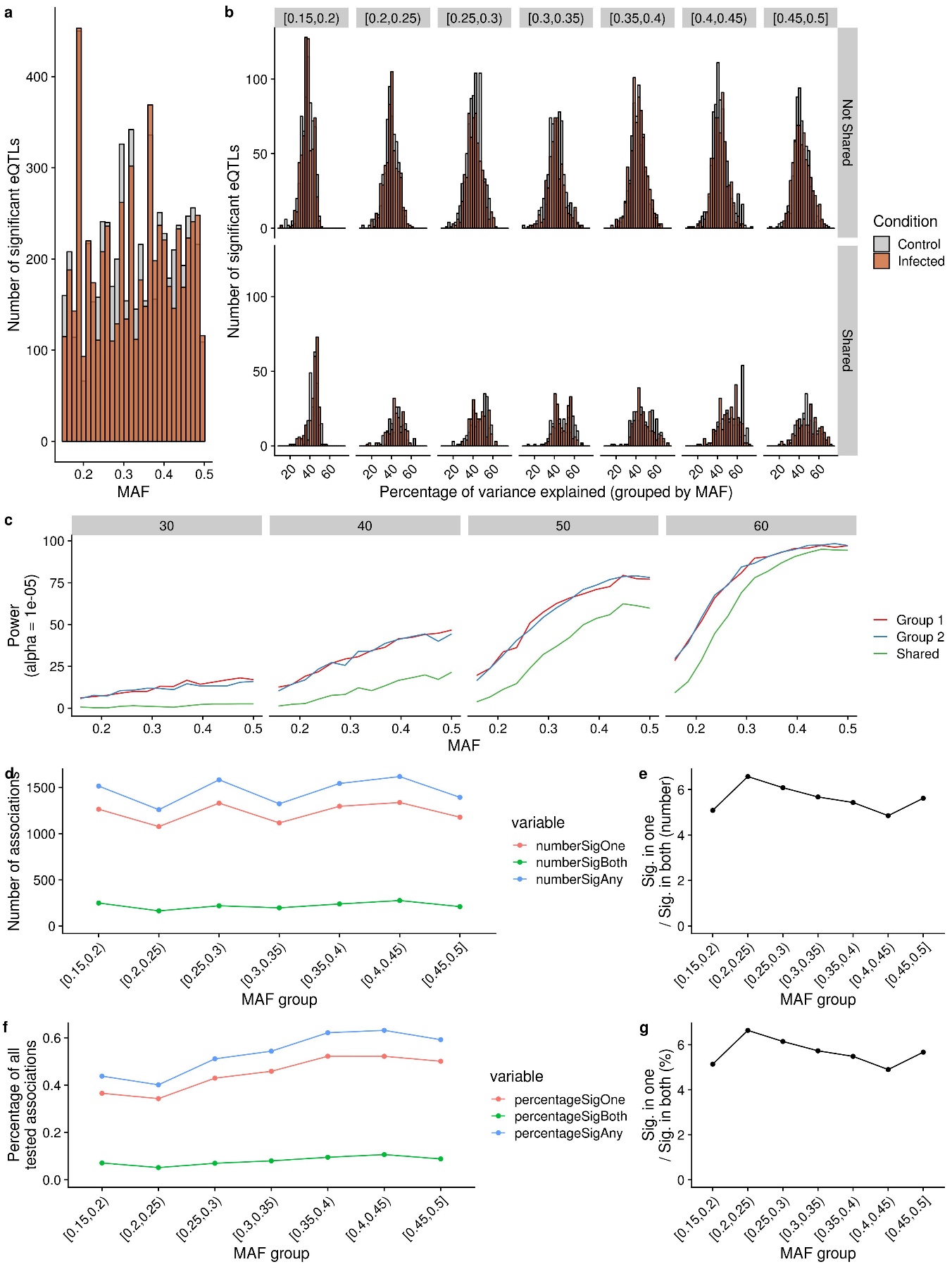


# Fig S3: Relationship between minor allele frequency and condition-specific local-eQTLs.

1. Histogram of significant *local-*eQTLs binned by minor allele frequency (MAF) and coloured by condition.
2. Histogram showing the contribution to variance in gene expression by genotype at significant *local-*eQTLs. The data are stratified according to condition, MAF, and whether the *local*-eQTL is shared. The same covariates are considered as in the *local-*eQTL analysis.
3. Simulation study under idealized conditions for a range of MAFs and contributions to variance in line means. For each MAF and expected contribution to gene expression level by the genotype, 1000 permutations were performed as in (2). To simulate gene expression, a random vector I with values 0 or 1 (representing genotypes), was created. To this vector, we then added a generated normal distribution (V_E_) that has a variance equal to the remaining variance such that V_E_ = ((100 – PerVar)/PerVar)*V_I_, where V_I_ = Var(I) and Pervar = contribution to variance in line means. The same analysis was performed in pairs (termed Group1 and Group2) that share all parameters but differ in the randomly-generated phenotypic variation that corresponds to V_E_. The Shared group in green corresponds to the power to detect a true shared eQTL and represents permutations where the eQTL is recovered in the pair.
4. Significant *local*-eQTLs were binned by minor allele frequency groups (MAF) and classified as significant in any (numberSigAny), only one (numberSigOne), and both conditions (numberSigBoth). Here, MAF is the number of strains carrying the less-represented allele in the 38 samples divided by 38.
5. Ratio of the number of *local*-eQTLs that are significant in one condition versus those that are shared as a function of MAF.
6. The same values in (a) represented as the percentage of all tested variants in each MAF group.
7. Ratio of the values in (c).


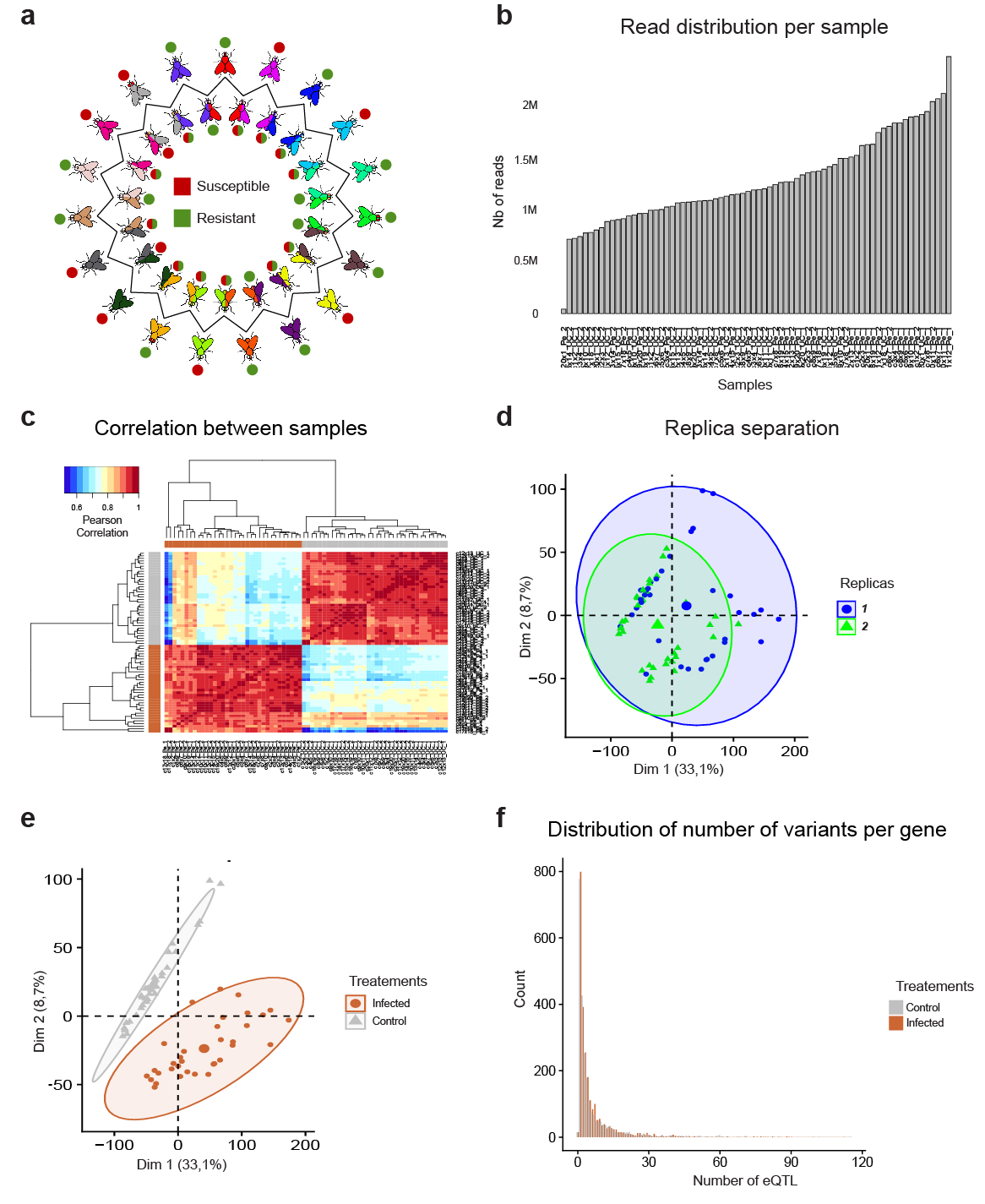


# Fig S4: Quality control of BRB-seq libraries

1. Repartition of resistant and susceptible lines in the round robin scheme.
2. Distribution of reads in each library. The left-most library was subsequently dropped from the analysis.
3. Correlation matrix between samples computed on the count matrices displays good separation between treatments.
4. Principal component analysis of gene expression from F1 lines, showing no major batch effect. Treatment-based PCA showing separation between different conditions.
5. Principal component analysis of gene expression from F1 lines, showing a separation between control and infected conditions.
6. Distribution of number of variants per gene.


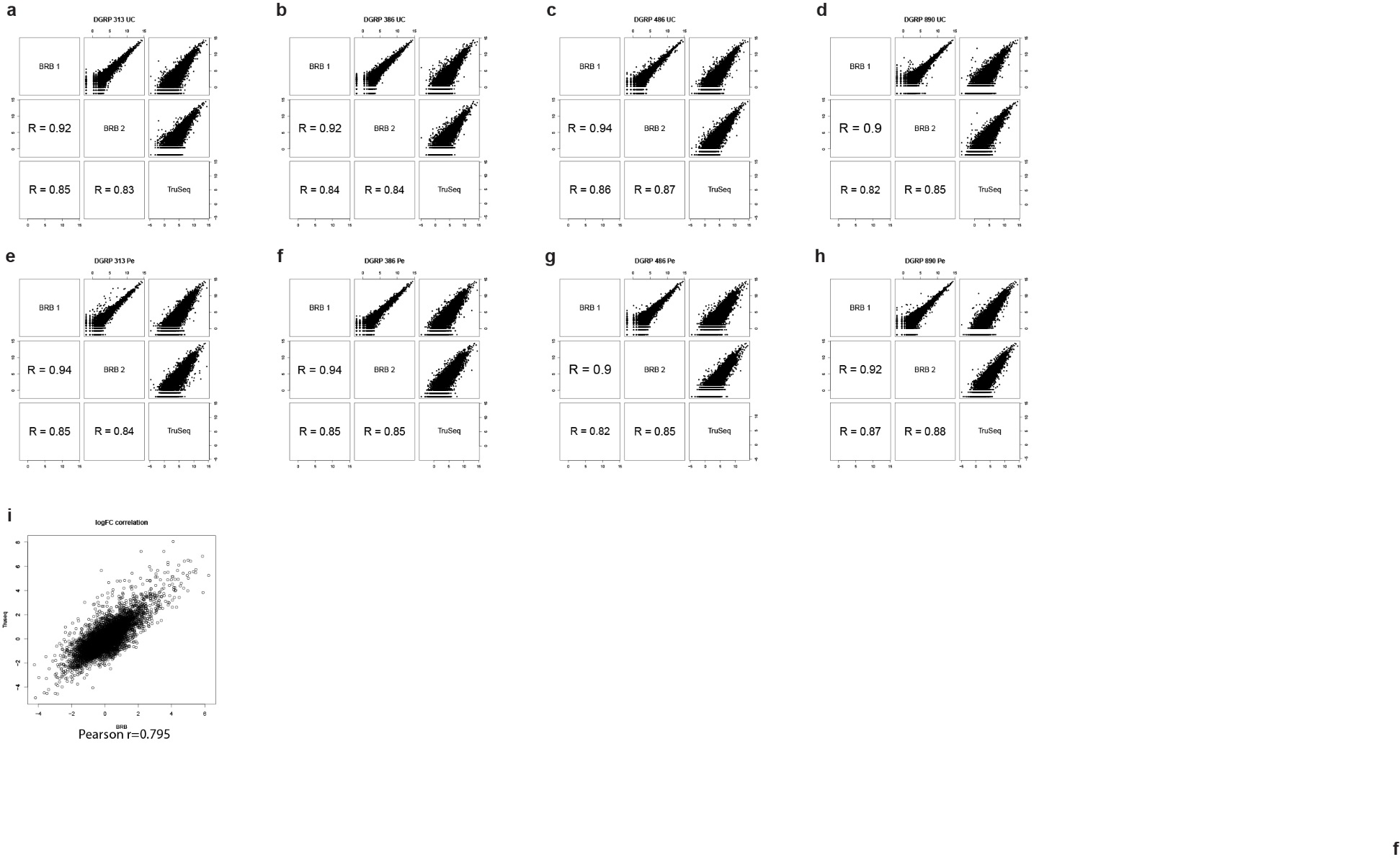


# Fig S5: Comparison between TruSeq and BRB-seq libraries

1. to (d) Correlation between libraries for the control condition. Each panel shows the correlation between the two BRB-seq libraries (BRB 1 and BRB 2) and the TruSeq library (TruSeq) for a particular DGRP line (from a to d: DGRP 313, DGRP 386, DGRP 486, and DGRP 890). Each matrix displays the correlation plot (upper triangle) and the Pearson correlation coefficient (bottom triangle).
2. to (h) Similar analysis as shown in panel (a) to (d) but for the infected condition (from e to h: DGRP 313, DGRP 386, DGRP 486 and DGRP 890). Correlation between gene expression fold change derived from the TruSeq experiment (y-axis) and from the BRB-seq experiment (x-axis). The Pearson correlation coefficient is provided under the plot.


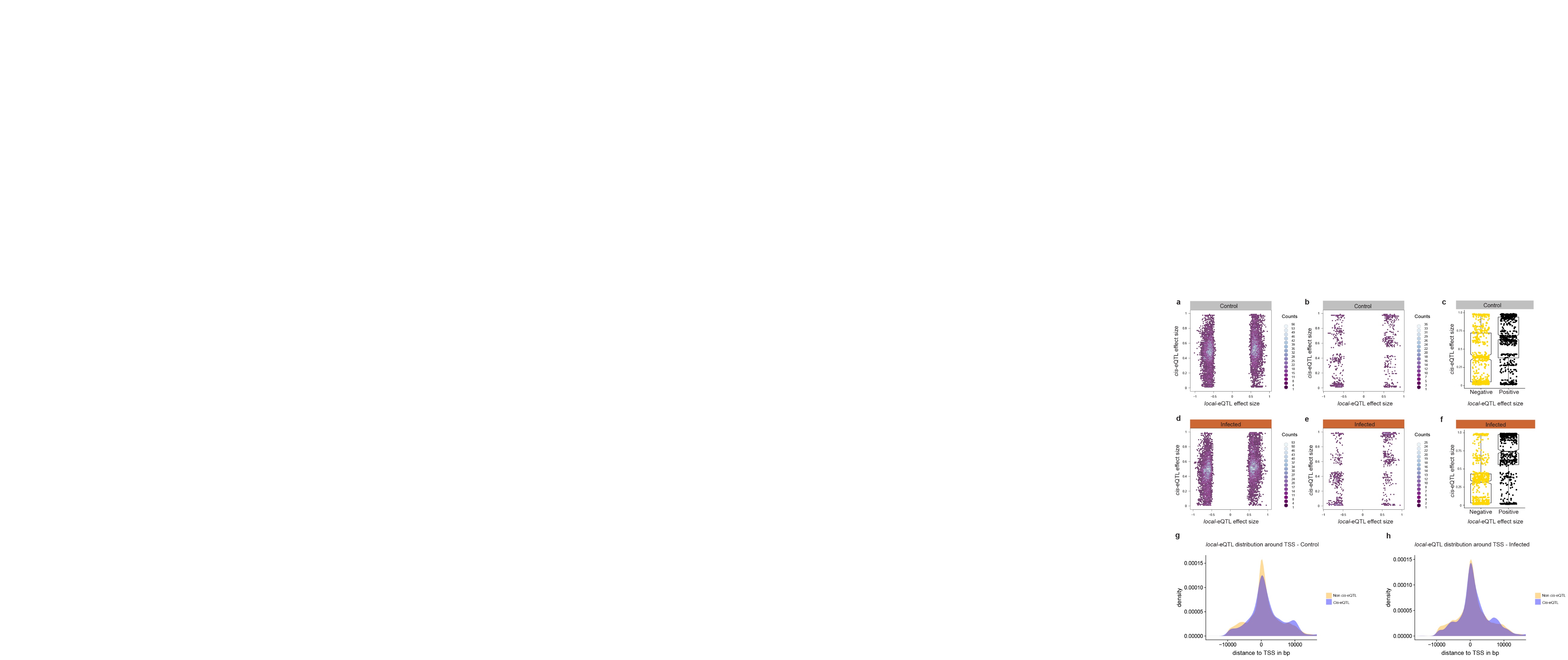


# Fig S6: Comparison of measured *local-*eQTL effect size and *cis-*eQTL effect size

1. Correlation between all *local*-eQTL effect size computed by Matrix-eQTL (x-axis) and *local-*eQTL effect size computed by allele specific expression (y-axis) showing a poor overall correlation in the control condition.
2. and (c) Correlation between *local*-eQTL effect size (x-axis) and *cis­-*eQTL (y-axis) effect size showing that a *local-*eQTL having a *cis*-effect will tend to replicate the effect size direction measured by Matrix-eQTL in the control condition.
3. Correlation between all *local*-eQTL effect size computed by Matrix-eQTL (x-axis) and *local-*eQTL effect size computed by allele specific expression (y-axis) showing a poor overall correlation in the infected condition.
4. and (f) Correlation between *local*-eQTL effect size (x-axis) and *cis­-*eQTL (y-axis) effect size showing that a *local-*eQTL having a *cis*-effect will tend to replicate the effect size direction measured by Matrix-eQTL in the infected condition.
5. This effect is stronger for the infected (panel (e) and (f)) compared to the control condition (panel (b) and (c)).
6. Distribution of non *cis-* and *cis-*eQTLs around the TSS for the control condition.
7. Distribution of non *cis-* and *cis-* eQTLs around the TSS from the infected condition.

**
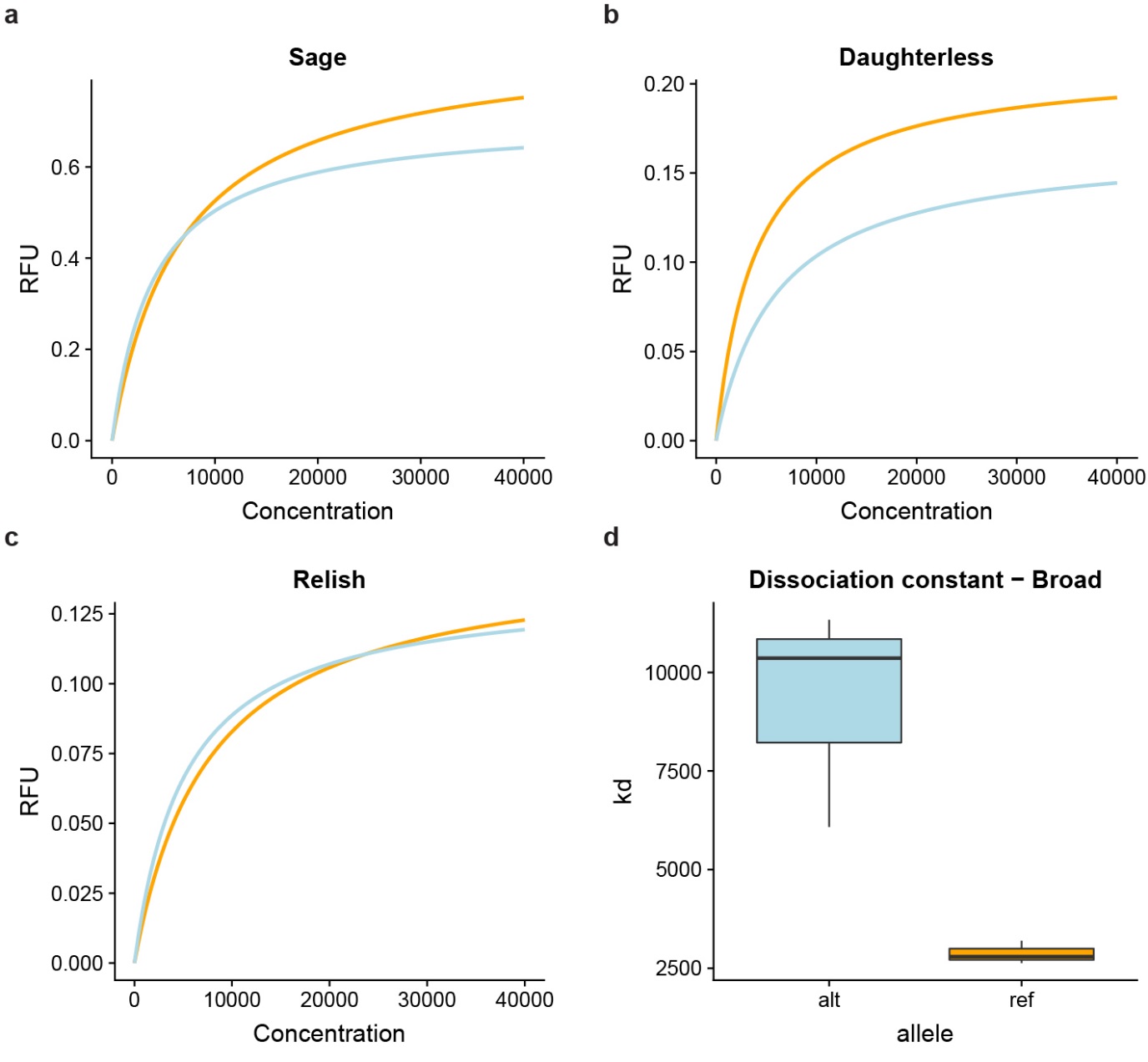
**

# Fig S7: MITOMI analysis of distinct TFs associated with the *ntc* locus

1. Measure of the binding affinity between Sage and the reference or alternate allele in one replica as measured by MITOMI (3,4).
2. Measure of the binding affinity between Daughterless and the reference or alternate allele in one replica as measured by MITOMI (3,4).
3. Measure of the binding affinity between Relish and the reference or alternate allele in one replica as measured by MITOMI (3,4).
4. Difference in the inferred dissociation constant (Kd) between the *Broad* alternate (blue) and reference (orange) alleles in three replicas.

**References**

1. Bou Sleiman MS, Osman D, Massouras A, Hoffmann AA, Lemaitre B, Deplancke B. Genetic, molecular and physiological basis of variation in Drosophila gut immunocompetence. Nat Commun [Internet]. 2015;6:7829. Available from: http://www.nature.com/doifinder/10.1038/ncomms8829

2. Long AD, Macdonald SJ, King EG. Dissecting complex traits using the Drosophila Synthetic Population Resource. Trends Genet [Internet]. 2014;30(11):488–95. Available from: http://dx.doi.org/10.1016/j.tig.2014.07.009

3. Isakova A, Groux R, Imbeault M, Rainer P, Alpern D, Dainese R, et al. SMiLE-seq identifies binding motifs of single and dimeric transcription factors. Nat Methods. 2017;14(3):316–22.

4. Maerkl SJ, Quake SR. A Systems Approach to Measuring the Binding Energy Landscapes of Transcription Factors. Science (80- ). 2007;315(5809):233–7.
