# Supplementary material for: cis-regulatory variation modulates susceptibility to enteric infection in the Drosophila genetic reference panel
Source: Genome Biol. 2020 Jan 17;21:6. doi: 10.1186/s13059-019-1912-z (PMC6966807; doi:10.1186/s13059-019-1912-z)
Supplement: Supplementary file 2 — Additional file 2. Review history. [file 13059_2019_1912_MOESM2_ESM.docx]

Review History

**First round of review**

**Reviewer 1**

**Are you able to assess all statistics in the manuscript, including the appropriateness of statistical tests used?**

Yes, and I have assessed the statistics in my report.

**Comments to author:**

TITLE: cis-regulatory variation modulates susceptibility to enteric infection in the Drosophila Genetic Reference Panel

AUTHORS: Frochaux, Sleiman, Gardeux, Dainese, Hollis, Litovchenko, Braman, Osman, Deplancke

SUMMARY OF MANUSCRIPT: The authors carry out an RNAseq study using inbred lines of the DGRP panel, which they previously employed to carry out a GWAS for resistance to P. entomophila. Here, using a set of 38 lines, half of which are relatively resistant and half of which are relatively susceptible to infection, they carry out gut-specific RNAseq on naive genotypes and genotypes exposed to the bacterium. Only 1 gene comes out as differentially-expressed between the resistant and susceptible classes, and only in the infected treatment (not in the control). Much of the second half of the manuscript attempts to uncover more detail about the effect of the ntc gene on infection response; Examining the effect of an ntc null (which reduces resistance), examining expression of Diptericin in ntc mutant/control animals, and examining via an in vitro technique whether polymorphisms up- and downstream of the gene that are predicted eQTL impact binding of transcription factors. Their data support the idea that the Broad transcription factor affects expression of ntc differentially based on an upstream cis polymorphism. The first half of the paper is a more of a broad examination of eQTL. The authors identify a few thousand eQTL in their initial inbred line study, arguing that the minority of the eQTL that are shared between naive and infection conditions is due to different mechanisms of action in the two treatments. They then use a crossing design to generate F1s between DGRP strains, and use a different RNAseq methodology to examine allelic imbalance (where alleles in a heterozygote are differentially expressed). While some of the eQTL mapped in the inbred study are replicated in the cross design, most are not, and the authors imply that the eQTL mapped in the inbred study are therefore not true cis-eQTL. And they present some evidence that some of the inbred line study eQTL are in fact trans-eQTL.

SUMMARY OF REVIEW: There is a lot to like in this manuscript, from the genomewide examination of the regulatory response to infection in a well-used Drosophila mapping resource (thus providing an interesting/valuable dataset to the large community working on infection/resistance/microbiome in the fly model system), to a more detailed examination of the effect of a single gene - ntc - that comes out of their differential expression analysis as a novel candidate underlying resistance. However, I have a number of recommendations that might enhance the work, or at least its presentation, and a couple of criticisms about the interpretation of the results (particularly the eQTL screens) that the authors might consider.

(1) Presentation.

The paper is somewhat of a difficult read since it includes questions addressed at the broad genomewide scale, and questions that are more specifically oriented towards understanding the effect of a single gene. Its possible (maybe likely) that it is challenging to bring these parts together more smoothly, but right now the two ideas don't fit together that well for me. Obviously the authors should present their work in the way they feel is most appropriate, but I might suggest that the authors revise the order of the materials to: (i) naive vs infection differential expression, (ii) inbred line eQTL mapping (refering to the ntc eQTL more directly), (iii) the cross design eQTL mapping (again with a greater focus on ntc), (iv) the more ntc-specific mechanistic material. By continuing to focus throughout on ntc it might help lead the reader through the material, and bring the sections together better.

Regardless of whether the authors choose to alter the broad format of the manuscript, I would ask them to review my point-by-point notes below since there are an array of places in the figures/text that really need to be edited to be clearer.

(2) More appropriate analysis.

I was confused why the authors were considering the naive and infection treatment data separately in the eQTL analysis: They are doing the same genotype-to-expression association test within each treatment. It would seem more appropriate to include "treatment" in the model and carry out one test per polymorphism, and get an actual estimate of the effect of treatment. Perhaps there is some technical reason this can't be carried out, but its a more an appropriate analysis I feel.

On a different, but related point, I don't think there is sufficient information presented in the "methods" section to allow a reader to recapitulate the authors results. For instance, "We performed separate analyses for each infected condition using Matrix-eQTL" (line 533) doesn't tell me what the model actually was for the eQTL mapping - i.e., how did they run the software? There are numerous points in the "methods" where I had similar questions. I would recommend that the authors add detail to the "methods" throughout, and - where possible - provide actual code for their analyses as supplementary material.

(3) Power and interpretation of results.

A principal contention of the inbred line eQTL work is that there are lots of condition-specific eQTL (i.e., eQTL only found either in the naive or in the infection treatment, but not in both.) While the authors present a reasonable biological argument for this result, the interpretation is missing discussion of power. This is key. If the authors have (say) >90% power to detect eQTL of some effect in each treatment, then limited sharing does indeed tell you something biologically interesting. If, however, power is <50% (for instance), then one doesn't expect much sharing, purely due to technical effects. Even given that cis-eQTL often have large effects in published studies, 38 lines is relatively few, so I doubt power pushes towards 90% for eQTL of realistic effect size. I think the authors need to estimate power, add interpretive text to the manuscript (assuming that power is a concern), and perhaps carry out analyses to suggest their biological interpretation is still supported (e.g., if you restrict analysis to really large effect eQTL - which there is the most power to find - and one still sees a lack of sharing, then there is something biologically-interesting going on as they currently imply.)

Similarly, the authors contend that their inability to recapitulate inbred line eQTL as eQTL in the crossing experiment suggests that "local" eQTL from the inbred line experiment are typically not "cis" eQTL in the crossing/allelic imbalance experiment. Again, here one has two methods that find eQTL, and one is asking whether in each method the same eQTL is pulled up. Power is again relevant as to whether one can find overlapping eQTL if they do exist, so my concerns from above are relevant here also. Furthermore this comparison is affected not only by power, but by the fact that the original data was collected in different ways in the two experiments (TruSeq versus BRBseq, along with - if I'm reading the "methods" correctly - a host of differences in how the tissue/RNA samples themselves were collected). Its not clear to me what the expected correlation is between read counts at all the fly genes between the two techniques even if they are carried out on the exact same RNA sample (there is no data on this in the manuscript, and the authors may need to generate it if they wish to formally compare the datasets.) If the correlation is less than 100%, this - in addition to power - will also impact the expected level of eQTL overlap between studies. I think the authors need to do a lot of work in these comparative sections to highlight the technical differences in the two experiments, and highlight the potential technical reasons for the lack of overlap in addition to their biological reasons.

Finally, only 1 gene is found to be differentially expressed between the RILs that (in their previous phenotypic screen) are classified into resistant and susceptible groups. The authors have a reasonable biological explanation for this. But again, I think they're missing a simpler explanation for the result: Maybe gut-specific expression at 4 hours has very little to do with whole-animal resistance. In order words, there might be some regulatory correlate of resistance, but not one that is evident at the timepoint the authors chose. Some discussion of this possibility is warranted, or presentation of data/results that suggests this _is_ a critical period during infection, and therefore that their current interpretation is plausible.

POINTS:

(1) Lines 160-161: How are you defining "genes expressed in the gut"?

(2) Line 179: Clarify that the polymorphism that harbors the eQTL is actually in an Atf2 DNA binding site.

(3) Line 242-244: It took me ages to understand what this figure was telling me. I would advocate for a better description in the text leading the reader through this figure.

(4) Line 251: Define what 'beta' is.

(5) Line 315: Avoid use of "linkage groups" in this context, maybe just "SNP clusters"?

(6) Lines 318-320: Needs a statistical test to back up this statement.

(7) Lines 332-337: A couple of statements need statistical tests to back them up.

(8) Lines 331-332: What is the evidence animals containing balancers are "weaker" than those without? Is the contention that the yellow line in the bottom panel of 4c different from the yellow line in the top panel? Clarification, and a statistical test is valuable here.

(9) Lines 332-334: The ntc mutant tested is a null, so this RT-qPCR experiment isn't really surprising, right? I sort of think that the real story in this section is that Diptericin is down when you have an ntc null, since the authors appear to be using Diptericin expression as a "readout" for immune response. Some clarification here could be valuable.

(10) Line 354: There is nothing about a 10^-5 P-value threshold that is "stringent" in the context of a GWAS using >2 million variants, despite the pervasiveness of this threshold in papers doing GWAS in the DGRP. It would be more useful to talk about the FDR of these variants in the original GWAS.

(11) Lines 377 and 380: Were t-tests used for these read count comparisons? Not something that uses counts directly?

(12) Line 447: Diptericin expression isn't "absent" just lower in the genotype carrying the ntc null. I also realized here that the gene symbol for Diptericin isn't "Dipt", and remembered there are 2 Diptericin genes in D. mel (DptA and DptB). Change the symbol to the correct gene ID and clarify which gene is being tested.

(13) Line 459: I might perhaps be a little more circumspect here; the binding site test was in vitro. Unless there is some other evidence for in vivo binding of Broad to this position.

(14) Line 502: Truseq libraries really started with 1ng total RNA? Seems low given you have total RNA from several guts, and that can get quite a lot of total RNA. Was there a reason to start with this little?

(15) Line 510-11: Should release the custom (presumably?) code used to do this.

(16) Para starting on Line 517: This paragraph is a bit confusing. My principal question is whether the RNAseq data from those samples collected for a previous study were combined with new data collected for this study for the eQTL analysis presented. Or was the comparison between studies outlined here the only time the old data was used?

(17) Line 524: What is meant by "class" ?

(18) Line 533: Was a kinship matrix used in the analysis? Did you account for Wolbachia infection? inversion status? Often kinship/relationship matrices are important in GWAS, and wolbachia/inversion status are routinely accounted for in DGRP GWAS since they can have appreciable effects.

(19) Line 534: "5" what?

(20) Line 718: I have no idea what "semantic similarity" is, or how its calculated. Can this be clarified?

(21) Line 768: "Fold change" in what?

(22) Lines 773 and 775: The gene symbol is "RpL32".

(23) Figure 1c: What does the red "Random" line represent?

(24) Figure 1d: I have very little idea what this plot is telling me. Is there something about the positioning of the bubbles in X/Y space that informs me about how related the GO categories are? What do the log10_p_value values mean?

(25) Figure 2b: What does "significant" mean here? Significant at what level? (5% and 10% are variously used in the text.)

(26) Figure 3: This should be in the text, but reading the figure is where it occurred to me; It would valuable to give the effect of the ntc gene on expression change between resistance and susceptible groups.

(27) Figure 4: This figure is extremely challenging to interpret. Briefly: What do the colors refer to in the left set of boxplots in 4a? What do the letters mean below the 4b plots (can the codes be written out for clarity)? Avoid using "Ctrl" and "Test" in 4d/e - use the actual genotype names for clarity. For 4d/e I would advocate putting the "infected" bars all together using the same axes, and then the "naive" bars all together using different axes. Right now there is no way to see if there is anything interesting happening in the naive condition given the y-axis relative to the bar height.

**Reviewer 2**

**Are you able to assess all statistics in the manuscript, including the appropriateness of statistical tests used?**

Yes, and I have assessed the statistics in my report.

**Comments to author:**

The study combines Drosophila genomic resources and extensive transcriptional data from a well thought through experiment in a very smart way to demonstrate a link between a cis-regulatory variant in a transcription factor binding site and the expression of a gene (ntc). Furthermore, the authors convincingly show in functional experiments that the expression of that gene indeed affects infection susceptibility. Overall, the conclusions are well supported by the data and this is a very substantial piece of work that will serve as an example for more work that we will see in the future. The study is original and timely and should be a good fit for Genome Biology.

What is currently missing is accessibility to the raw data and to the analysis scripts that the authors used. This is required to make the study fully reproducible, adhere to current standards, and to make it possible for me to thoroughly evaluate the applied procedures, which I consider a part of my job as a reviewer. On a similar note, the materials and methods section needs work. For example, the description of RNA extraction in l496-499 is a stub. It is not even mentioned what RNA was extracted from. Confusingly, there is a much longer version of the RNA extraction procedure on a different experiment starting in l 543. Another example is the PCA in starting in line 528. It is not even said what the subject of PCA was. Where are the details for the TF binding site enrichment analysis?

Furthermore, I think the manuscript could be improved, regarding structure. If I understood correctly, there are two major transcriptome data sets: one for the eQTL analysis and one for the allele specific expression (ASE) analysis. Currently it seems to me that the manuscript alternates between them. The first results section deals with the eQTL dataset, the second with ASE, then the third comes back to the eQTL data set? Is this correct? It is not clearly mentioned what the data set is that the third results section starting at line 275 deals with. I first thought it must be the ASE data set because this section comes right after the ASE analysis and starts with PCA of gene expression. Finding out that it was probably the eQTL data set took me a while and disrupted coherence and readability. An easy solution to this might be to mention the switch back in data sets at the beginning of the paragraph.

A suggestion for the author's consideration:

A suggestion for a much more natural solution would be to start the results section with the finding from the eQTL data set that only ntc is differently expressed and then focus on how first the eQTL and then the additional ASE data were used to track down the regulatory variants. I think this approach would be logically more coherent and focused. Furthermore, I am not fully convinced that the eQTL and ASE results as such deserve the spotlight position they currently occupy. This is because there is no correlation between the results (Figure 2D and 2E) and a large proportion of loci could neither be assigned cis nor trans affects. Therefore, other parts of the manuscript are much more exciting. Although I fully respect the importance of these analyses and the work that went into it, the detailed description and interpretation of the results that seems speculative sometimes (l398-426) was distracting from the main message that I thought was that the authors provide a great example of how to establish the connection between a regulatory variant and an important phenotype. Hence, I think a focus on how eQTL and ASE data were leveraged in the context of this central theme could benefit readability and focus of manuscript.

Minor comments:

l43 It is not clear what is meant by "channeled through distinct molecular layers"

l79 There is a citation that was not converted to the numbering format and that is not listed in the references (Brem...)

l108 I think the choice of Pseudomonas entomophila needs some explanation for all readers not familiar with your previous work. It should be clarified at least that it is a pathogen that causes disease when ingested by the flies. Because the authors write in the discussion (l459) that they are looking an "ecologically-relevant trait" by analyzing immunocompetence towards P.e., they might also consider to mention here that Pseudomonas occurs in natural D. melanogaster population quite frequently (e.g Staubach et al 2013 or Wang & Staubach 2018).

l133 It is not very well explained what is meant by individualized genomes. This should be better explained in the methods section. Does it mean you are just keeping homozygous SNPs?

l267 there is no figure 3f

l275 It is not "few genes" but a single gene (ntc), or am I getting something wrong?

l285-295 could go to the discussion

l306-309 I do not understand

l313/Fig3c I would really like to see the data on the expression levels of ntc in a plot to judge the difference between susceptible and resistant lines since much of the paper hinges on this result.

**Authors Response**

**Point-by-point responses to the reviewers’ comments:**

Reviewer #1:

SUMMARY OF MANUSCRIPT: The authors carry out an RNAseq study using inbred lines of the DGRP panel, which they previously employed to carry out a GWAS for resistance to P. entomophila. Here, using a set of 38 lines, half of which are relatively resistant and half of which are relatively susceptible to infection, they carry out gut-specific RNAseq on naive genotypes and genotypes exposed to the bacterium. Only 1 gene comes out as differentially-expressed between the resistant and susceptible classes, and only in the infected treatment (not in the control). Much of the second half of the manuscript attempts to uncover more detail about the effect of the ntc gene on infection response; Examining the effect of an ntc null (which reduces resistance), examining expression of Diptericin in ntc mutant/control animals, and examining via an in vitro technique whether polymorphisms up- and downstream of the gene that are predicted eQTL impact binding of transcription factors. Their data support the idea that the Broad transcription factor affects expression of ntc differentially based on an upstream cis polymorphism. The first half of the paper is a more of a broad examination of eQTL. The authors identify a few thousand eQTL in their initial inbred line study, arguing that the minority of the eQTL that are shared between naive and infection conditions is due to different mechanisms of action in the two treatments. They then use a crossing design to generate F1s between DGRP strains, and use a different RNAseq methodology to examine allelic imbalance (where alleles in a heterozygote are differentially expressed). While some of the eQTL mapped in the inbred study are replicated in the cross design, most are not, and the authors imply that the eQTL mapped in the inbred study are therefore not true cis-eQTL. And they present some evidence that some of the inbred line study eQTL are in fact trans-eQTL.

SUMMARY OF REVIEW: There is a lot to like in this manuscript, from the genome-wide examination of the regulatory response to infection in a well-used Drosophila mapping resource (thus providing an interesting/valuable dataset to the large community working on infection/resistance/microbiome in the fly model system), to a more detailed examination of the effect of a single gene - ntc - that comes out of their differential expression analysis as a novel candidate underlying resistance.

*We thank the reviewer for his/her appreciation of our work.*

However, I have a number of recommendations that might enhance the work, or at least its presentation, and a couple of criticisms about the interpretation of the results (particularly the eQTL screens) that the authors might consider.

(1) Presentation.

The paper is somewhat of a difficult read since it includes questions addressed at the broad genomewide scale, and questions that are more specifically oriented towards understanding the effect of a single gene. Its possible (maybe likely) that it is challenging to bring these parts together more smoothly, but right now the two ideas don't fit together that well for me. Obviously the authors should present their work in the way they feel is most appropriate, but I might suggest that the authors revise the order of the materials to: (i) naive vs infection differential expression, (ii) inbred line eQTL mapping (refering to the ntc eQTL more directly), (iii) the cross design eQTL mapping (again with a greater focus on ntc), (iv) the more ntc-specific mechanistic material. By continuing to focus throughout on ntc it might help lead the reader through the material, and bring the sections together better.

Regardless of whether the authors choose to alter the broad format of the manuscript, I would ask them to review my point-by-point notes below since there are an array of places in the figures/text that really need to be edited to be clearer.

*We thank both reviewers for pointing out that the flow of the article could be enhanced. Following their advice, we have substantially reshuffled the different parts of our manuscript such that we now present the differential gene expression analysis first, then the eQTL study followed by the allele-specific expression analysis, each time using ntc as the guiding thread throughout the manuscript for improved flow and clarity.*

(2) More appropriate analysis.

I was confused why the authors were considering the naive and infection treatment data separately in the eQTL analysis: They are doing the same genotype-to-expression association test within each treatment. It would seem more appropriate to include "treatment" in the model and carry out one test per polymorphism, and get an actual estimate of the effect of treatment. Perhaps there is some technical reason this can't be carried out, but it’s a more an appropriate analysis I feel.

*We thank the reviewer for pointing this out. We had performed this early on in the analysis and decided against doing this for the following reasons:*

*- Interpretability: it is easier to interpret a simpler model with no additional covariates and no need to incorporate interaction terms.*

*- Statistical concerns: given that each strain is listed twice (once per condition), we would need to consider the strain, and then the choice would be whether to take strain as random effect, or just duplicate the genotypic PC (genetic relatedness) and all other covariates. Despite these concerns, we have performed the analysis with the full dataset and identified a very high number of eQTLs (~10 times more than a single condition – containing 90 and 82% of the control and treated eQTLs, respectively) at the same significance thresholds. However, we believe this is an artificial inflation in the p-values due to the correlation between two samples of the same strain and the fact that we are using inbred lines.*

*- For reasons similar to those stipulated above, studies that also feature comparative eQTL analyses tend to also treat different treatments/timepoints separately (see e.g. Strober et al., Science, 2019).*

*For those reasons, we preferred the simpler approach over the complex (and in our opinion more biased) one in the manuscript. Please note however that we have nevertheless updated the model to incorporate additional parameters such as kinship and Wolbachia status, as requested by the reviewer in point 18 below. This also resulted in a relatively minor alteration of the final eQTL dataset, as discussed in more detail in our response to point 18. We also added a clarification in the manuscript (lines 221-226).*

On a different, but related point, I don't think there is sufficient information presented in the "methods" section to allow a reader to recapitulate the author’s results. For instance, "We performed separate analyses for each infected condition using Matrix-eQTL" (line 533) doesn't tell me what the model actually was for the eQTL mapping - i.e., how did they run the software? There are numerous points in the "methods" where I had similar questions. I would recommend that the authors add detail to the "methods" throughout, and - where possible - provide actual code for their analyses as supplementary material.

*We agree that the code and data must be public. A repository was created and can be found on our lab github (https://github.com/DeplanckeLab/Frochaux_BouSleiman_2020). Furthermore, we have significantly expanded the Methods Section for improved clarity (Lines 576-583, 591-601, 614-648).*

(3) Power and interpretation of results.

A principal contention of the inbred line eQTL work is that there are lots of condition-specific eQTL (i.e., eQTL only found either in the naive or in the infection treatment, but not in both.) While the authors present a reasonable biological argument for this result, the interpretation is missing discussion of power. This is key. If the authors have (say) >90% power to detect eQTL of some effect in each treatment, then limited sharing does indeed tell you something biologically interesting. If, however, power is <50% (for instance), then one doesn't expect much sharing, purely due to technical effects. Even given that cis-eQTL often have large effects in published studies, 38 lines is relatively few, so I doubt power pushes towards 90% for eQTL of realistic effect size. I think the authors need to estimate power, add interpretive text to the manuscript (assuming that power is a concern), and perhaps carry out analyses to suggest their biological interpretation is still supported (e.g., if you restrict analysis to really large effect eQTL - which there is the most power to find - and one still sees a lack of sharing, then there is something biologically-interesting going on as they currently imply.)

*We agree with the reviewer that power may be a concern and thus to what extent are condition-specific eQTLs a mere reflection of lack of power? Addressing this formally and without assumptions on the distributional properties of gene expression, allele frequencies of variants, linkage between variants, and varying densities of variants in the genic windows selected for eQTL analysis is however far from a trivial task. Nevertheless, we have developed an indirect strategy that we hope will satisfy the reviewer. In this strategy, based on using our results without further assumptions, we capitalize on the known relationship between “power to detect an effect” on the one hand and “minor allele frequency (MAF) of variants” on the other. Here, we calculated MAF as the number of reference (or variant) homozygous lines (depending on which is less represented) divided by the number of lines (38). Please note that we tested for associations only when there were at least 6 lines with the MAF ~ 15.7%. The expectation is that the higher the MAF, the higher the power to detect a true eQTL and therefore the lower the propensity to detect false-positives that we may be falsely assign as condition-specific eQTLs. As for eQTLs that are shared, the case is strong for them being true eQTLs. So if we compare the number of associations that are significant in only one condition to those that are shared as a function of MAF, we would expect relatively more that are shared as we approach 0.5 MAF. However, we do not see this trend (see new Supplementary Figure 3). We binned significant associations by MAF ranges and counted the number of associations that are significant in any (numberSigAny), only one (numberSigOne), and both conditions (numberSigBoth). The number appears to be flat as MAF increases. If we take the same numbers as a proportion of total tested variant-gene pairs (panel b: percentageSigOne, percentageSigBoth, percentageSigAny), we can see that there is a trend of increase in both shared and condition-specific eQTLs. We interpret this increase in detected condition-specific eQTLs as an increase in power to detect true condition-specific eQTLs since this defies our expectation if these eQTLs were false positives. Panels b and d show the ratios of shared versus condition-specific eQTLs as a function of MAF. Here too, we can see that the ratio is hovering around 6. Based on these analyses, we conclude that the majority of condition-specific eQTLs are true positives, thus reflecting an interesting biological phenomenon as implied by the reviewer (Supplementary Figure 3, Lines 231-240).*

Similarly, the authors contend that their inability to recapitulate inbred line eQTL as eQTL in the crossing experiment suggests that "local" eQTL from the inbred line experiment are typically not "cis" eQTL in the crossing/allelic imbalance experiment. Again, here one has two methods that find eQTL, and one is asking whether in each method the same eQTL is pulled up. Power is again relevant as to whether one can find overlapping eQTL if they do exist, so my concerns from above are relevant here also. Furthermore this comparison is affected not only by power, but by the fact that the original data was collected in different ways in the two experiments (TruSeq versus BRBseq, along with - if I'm reading the "methods" correctly - a host of differences in how the tissue/RNA samples themselves were collected). Its not clear to me what the expected correlation is between read counts at all the fly genes between the two techniques even if they are carried out on the exact same RNA sample (there is no data on this in the manuscript, and the authors may need to generate it if they wish to formally compare the datasets.) If the correlation is less than 100%, this - in addition to power - will also impact the expected level of eQTL overlap between studies. I think the authors need to do a lot of work in these comparative sections to highlight the technical differences in the two experiments, and highlight the potential technical reasons for the lack of overlap in addition to their biological reasons.

*The similarity/correlation between TruSeq and BRB-seq datasets has been discussed in depth in the original paper(Alpern et al., 2019). However, we have also sequenced original DGRP lines along with our F1 flies. We were thus able to show that the two methods are correlated on the number of counts for each gene and on the fold change of differentially expressed genes, thus demonstrating that the BRB-seq dataset is able to recapitulate the original dataset at a raw level with similar read counts and information level with matching fold change. Moreover, since we use the F1 hybrids to measure a cis-effect, we feel that the need for 100% correlation is not even mandatory. We added this supplemental analysis (Lines 304-311) and a new figure (Supplementary Figure 5), highlighting these results.*

Finally, only 1 gene is found to be differentially expressed between the RILs that (in their previous phenotypic screen) are classified into resistant and susceptible groups. The authors have a reasonable biological explanation for this. But again, I think they're missing a simpler explanation for the result: Maybe gut-specific expression at 4 hours has very little to do with whole-animal resistance. In order words, there might be some regulatory correlate of resistance, but not one that is evident at the timepoint the authors chose. Some discussion of this possibility is warranted, or presentation of data/results that suggests this _is_ a critical period during infection, and therefore that their current interpretation is plausible.

*We thank the reviewer for this question. The fly gut’s response at the transcriptional level is very fast and some differences can be detected as early as the first 30 minutes of infection. By 6-8 hours, stem cell division and other processes kick in and expression results may be seriously biased by gross changes in gut morphology. By 16 hours (another commonly used timepoint in the field), some of the first deaths start to happen, and this is especially common in susceptible strains. So it is likely that resistant and susceptible strains may have very different transcriptomes at these later time points. We were rather interested in whether early changes in the transcriptome can inform about the outcome of the infection. For those reasons, we chose this timepoint as a convenient one at which most changes observed are (1) transcriptional in nature and (2) early enough to not be indirectly affected by resistance to infection. Furthermore, these timepoints were used in previous studies on oral infection (Buchon et al., Genes Dev., 2009; Chakrabarti et al., Cell Host & Microbe, 2012) using microarrays. We have explained the rationale underlying the selection of this time point now clearer in the revised manuscript (please see lines 134-135).*

POINTS:

(1) Lines 160-161: How are you defining "genes expressed in the gut"?

*We defined genes that are expressed in the gut as those that have at least 5 reads mapped in at least 38 samples (out of the 76). We added the definition to lines 244-248.*

(2) Line 179: Clarify that the polymorphism that harbors the eQTL is actually in an Atf2 DNA binding site.

*We thank the reviewer for pointing this out. In the earlier version of the manuscript, the analyses for transcription factor binding site enrichment were performed on windows +- 100 bp around each identified local-eQTL. The reasoning behind this choice being that the variants associated with the detected local-eQTLs may not be the causal variants. Hence, the question was whether there is an enrichment of binding sites in the vicinity of local-eQTLs. As for whether the eQTLs were actually overlapping an Atf2 site, this requires motif-scanning rather than motif-enrichment. This was never our intent, since we did not expect to find causal variants that way, yet we performed this analysis and found that very few variants overlap with a predicted motif binding site in general. For example, for Atf2 there were only 2 out of 98 predicted binding sites (raw p-value < 1e-5) in infection-specific local-eQTL windows.*

*However, given the updated local-eQTL list and with the goal of performing a more comprehensive analysis (compared to our original strategy based only on FlyFactorSurvey), we have now used the i-cisTarget web tool, which features a much more expansive motif database, but again using 201 bp windows around each eQTL as input based on the reasoning detailed above. We performed two analyses using regions from respectively the control- and infection-specific local-eQTLs, revealing a significant enrichment of GATA-like motifs in both datasets. Given the well-established role of GATA factors in gut development and homeostasis (Aronson, Stapleton, & Krasinski, 2014; Buchon et al., 2013), this result serves as a sanity check for our approach. Next, we performed a comparative analysis to determine differential motif enrichment. While Atf-2 enrichment did no longer emerge in the new data with fewer local-eQTLs, we found instead an interesting pattern showing that infection-specific local-eQTLs are differentially enriched for Forkhead box family motifs. In light of these more comprehensive results and recent findings showing that intestinal FoxO signalling is required for survival to oral infection in Drosophila (Fink et al., 2016), we believe that this new analysis is an important addition to our manuscript. We have therefore changed Figure 3d as well as the Abstract (line 49-50), Results (lines 258-268) and Materials and Methods accordingly (lines 641-648).*

(3) Line 242-244: It took me ages to understand what this figure was telling me. I would advocate for a better description in the text leading the reader through this figure.

*We apologize for the confusion and have now improved the discussion on this Figure. Please see lines 320-325.*

(4) Line 251: Define what 'beta' is.

*It is the effect-size computed by matrix-eQTL, a clarification was added to the revised manuscript (line 356).*

(5) Line 315: Avoid use of "linkage groups" in this context, maybe just "SNP clusters"?

*We agree and altered the description accordingly (Line 383).*

(6) Lines 318-320: Needs a statistical test to back up this statement.

*We have added the statistics from the differential expression results in the text to support our statement (lines 206-210).*

(7) Lines 332-337: A couple of statements need statistical tests to back them up.

*They have been added (Lines 189-198).*

(8) Lines 331-332: What is the evidence animals containing balancers are "weaker" than those without? Is the contention that the yellow line in the bottom panel of 4c different from the yellow line in the top panel? Clarification, and a statistical test is valuable here.

*We decided to remove this statement as it was based solely on our observation that ntc mutations tend to have stronger deleterious effects in lines with balancers (Lines 194-196).*

(9) Lines 332-334: The ntc mutant tested is a null, so this RT-qPCR experiment isn't really surprising, right? I sort of think that the real story in this section is that Diptericin is down when you have an ntc null, since the authors appear to be using Diptericin expression as a "readout" for immune response. Some clarification here could be valuable.

*The ntc mutant is indeed a null, however the genotypes tested are heterozygous for the mutant. There is still some ntc expression from the balancer or wild type chromosome. Thus, we feel that the amount of ntc expressed is relevant in the mutant analysis.*

(10) Line 354: There is nothing about a 10^-5 P-value threshold that is "stringent" in the context of a GWAS using >2 million variants, despite the pervasiveness of this threshold in papers doing GWAS in the DGRP. It would be more useful to talk about the FDR of these variants in the original GWAS.

*We agree with the reviewer and have thus removed the word “stringent”. However, we believe that the listing of p-values has still inherent value here as it provides the reader with an immediate read-out of association significance, which is why we propose to keep these values in the revised manuscript.*

(11) Lines 377 and 380: Were t-tests used for these read count comparisons? Not something that uses counts directly?

*We have now changed this analysis. We have now used the same approach as the one used for the general ASE analysis, i.e. a generalized mix model with binomial response on the read counts. We have however relaxed the stringent threshold used in our ASE analysis. Indeed, for the genome-wide analysis, we used a threshold defined as the minimum number of total reads superior to the maximum value between 6 or the 25th quantile of the total of reads assigned to the lineage lines in each sample (please see Methods). We used this threshold to eliminate false positives due to low read mapping issues, in particular for genes that are lowly expressed. However, since the lowest number of reads mapping to ntc was 35, implementation of this stringent threshold was no longer required. Hence, we applied the same generalized mixed model used for our general ASE analysis on all ntc heterozygous samples without any threshold, consistent with comparable analyses in previous studies (Castel, Levy-Moonshine, Mohammadi, Banks, & Lappalainen, 2015; McManus et al., 2010; Pinter et al., 2015). The new analysis can be found at lines 417-427.*

(12) Line 447: Diptericin expression isn't "absent" just lower in the genotype carrying the ntc null. I also realized here that the gene symbol for Diptericin isn't "Dipt", and remembered there are 2 Diptericin genes in D. mel (DptA and DptB). Change the symbol to the correct gene ID and clarify which gene is being tested.

*We apologize for this small oversight and have changed the description to DiptA in the whole manuscript.*

(13) Line 459: I might perhaps be a little more circumspect here; the binding site test was in vitro. Unless there is some other evidence for in vivo binding of Broad to this position.

*We have changed the description from “These results establish” to “These results suggest”, as our binding data is indeed based on an in vitro assay (Line 502).*

(14) Line 502: Truseq libraries really started with 1ng total RNA? Seems low given you have total RNA from several guts, and that can get quite a lot of total RNA. Was there a reason to start with this little?

*We apologize for the typo. We used 1 µg of total RNA, not 1 ng (Line 585).*

(15) Line 510-11: Should release the custom (presumably?) code used to do this.

*Please see our response to point 2.*

(16) Para starting on Line 517: This paragraph is a bit confusing. My principal question is whether the RNAseq data from those samples collected for a previous study were combined with new data collected for this study for the eQTL analysis presented. Or was the comparison between studies outlined here the only time the old data was used?

*The comparison with the other study is the only time the old data was used. We used these data as a quality control by demonstrating that the transcriptomic profiles of the re-sequenced lines were clustering closely with those from the original study (see Supplementary Figure 1a). We highlighted this in lines 141-147.*

(17) Line 524: What is meant by "class"?

*It means whether the line is resistant or susceptible. We have now added a clarification to the text (Line 610).*

(18) Line 533: Was a kinship matrix used in the analysis? Did you account for Wolbachia infection? inversion status? Often kinship/relationship matrices are important in GWAS, and wolbachia/inversion status are routinely accounted for in DGRP GWAS since they can have appreciable effects.

*In the initial submission, we did not include a kinship matrix or other parameters in our model as we preferred to keep the model simple, as discussed in more detail in our response to point 2 above. To address the reviewer’s question, however, we have now updated our analysis to include genetic relationship as well as Wolbachia infection. To take genetic relationship into account, we obtained the first three principal components using the SNPrelate R package. We did not take inversion status into account because it is highly correlated to the genotype PCs. We updated our analyses and results throughout. While we detected fewer eQTLs than before (6348 and 5904 versus 10575 and 9636 in the control and infection conditions respectively), 85% and 84% of the detected gene-variant associations overlapped with those obtained using a model without covariates in the control and infected condition, respectively, showing the robustness of our data. Importantly, the ntc local-eQTLs remained significant. The new method is now presented in lines 620-639.*

*Please find below a graphical representation of the comparison between the two approaches (withCov = with covariate, noCov = without covariate). As you can see, the beta (slope of the linear model) is well conserved. P-values are in general slightly lower when covariates are considered, as expected, but the general picture does not change.*

*Rebuttal Figure legend: Comparison of the summary statistics, notably the slope (beta) and the raw p-value, obtained from Matrix-eQTL using a model with covariates (three genotype principal components and Wolbachia status) and one without covariates. Each point is a significant association (a variant-gene pair) in the two scenarios. The control and infection results are in the upper and lower panels, respectively.*

*In sum, we thank the reviewer for his/her suggestion as we believe the incorporation of co-variates improved the quality of the data/manuscript. Please note that all downstream results were now also revised according to the new eQTL dataset. Specifically, we re-ran the GO enrichment analyses, updated the TF motif enrichment analyses and made these more comprehensive, and finally re-performed the ASE analysis. As for the ASE, we observed a decrease in the number of measured cis-effects.*

(19) Line 534: "5" what?

*We apologize for the confusion. The measure of MAF here is actually the number of lines carrying the less prevalent allele in the sampled strains. This translates to a minimum of 6/38 = 15.8%. This definition was added to the manuscript (Lines 624-626).*

(20) Line 718: I have no idea what "semantic similarity" is, or how its calculated. Can this be clarified?

*We used and cited a web tool called Revigo to simplify our resulting gene ontology results. Revigo itself builds up the method they call “simRel” to compare two GO terms to each other, based on the widely-used Resnik's and Lin's similarity measures. From the Revigo website: The simRel score ranges from 0 for terms that have no similarity to 1 for terms with maximum similarity.*

*We have included more details in the methods (lines 633-639), but feel that a more detailed description goes outside the scope of the manuscript and we therefore kindly refer the reader/reviewer to the respectively cited paper.*

(21) Line 768: "Fold change" in what?

*It is the fold change in Ntc expression, this was added at line 825.*

.

(22) Lines 773 and 775: The gene symbol is "RpL32".

*Thank you, we have corrected the name (Line 818 and 820).*

(23) Figure 1c: What does the red "Random" line represent?

*We apologize for not clarifying. We have now changed the figure legend of what is now Fig. 3b to the following:*

*(b) Metaplot of the location of local-eQTLs with respect to their associated genes’ transcription start sites (TSS). The grey, blue, and orange lines represent the control, shared, and infected conditions respectively. The black lines represent 100 subsamples of 5904 SNPs, the number of detected local-eQTLs in the infected condition, from all SNPs that were included in the association analyses, showing the random expectation of SNP location around a gene, irrespective of whether it is a local-eQTL (lines 832-838).*

(24) Figure 1d: I have very little idea what this plot is telling me. Is there something about the positioning of the bubbles in X/Y space that informs me about how related the GO categories are? What do the log10_p_value values mean?

*We have added the following to the legend of what is now Fig. 3c (Lines 845-848):*

*“The log10(p value) is the hypergeometric test FDR corrected p-value enrichment result. The X/Y space is the multidimensional scaling (MDS) of the pairwise semantic similarity measures (simRel). Closer terms in this 2D space imply closer GO terms (based on their similarity).”*

(25) Figure 2b: What does "significant" mean here? Significant at what level? (5% and 10% are variously used in the text.)

*Here, significant means that we detect a cis-effect at 5% FDR. We have changed the figure legend by replacing “significant” by “Infected/Control cis-effect” and “non-significant” by “Infected/Control No cis-effect” (Figure 4b).*

(26) Figure 3: This should be in the text, but reading the figure is where it occurred to me; It would valuable to give the effect of the ntc gene on expression change between resistance and susceptible groups. The description of ntc fold change and expression between resistant and susceptible are presented in Figure 4.

*It is now presented in the second part of our results (lines 206-210), figure 2d.*

(27) Figure 4: This figure is extremely challenging to interpret. Briefly: What do the colors refer to in the left set of boxplots in 4a? What do the letters mean below the 4b plots (can the codes be written out for clarity)? Avoid using "Ctrl" and "Test" in 4d/e - use the actual genotype names for clarity. For 4d/e I would advocate putting the "infected" bars all together using the same axes, and then the "naive" bars all together using different axes. Right now there is no way to see if there is anything interesting happening in the naive condition given the y-axis relative to the bar height.

*We have added an improved description of the color code to figure 4a (now figure 5a) and figure 4b (now figure 2d). For figures 4d and 4e (now figure 2b and 2e), we have added the genotype as suggested. However, we detected no expression of neither ntc or DiptA in the control condition. Indeed, in control conditions, the CT values were extremely high (thus reflecting very low expression). Therefore, rescaling the y axis would create confusion. We have also updated the legends for these panels (Lines 818-822 and 824-825).*

Reviewer #2:

The study combines Drosophila genomic resources and extensive transcriptional data from a well thought through experiment in a very smart way to demonstrate a link between a cis-regulatory variant in a transcription factor binding site and the expression of a gene (ntc). Furthermore, the authors convincingly show in functional experiments that the expression of that gene indeed affects infection susceptibility. Overall, the conclusions are well supported by the data and this is a very substantial piece of work that will serve as an example for more work that we will see in the future. The study is original and timely and should be a good fit for Genome Biology.

*We thank the reviewer for his/her appreciation of our work.*

What is currently missing is accessibility to the raw data and to the analysis scripts that the authors used. This is required to make the study fully reproducible, adhere to current standards, and to make it possible for me to thoroughly evaluate the applied procedures, which I consider a part of my job as a reviewer.

*We have made all of this material now publicly available as also detailed in our response to reviewer 1.*

On a similar note, the materials and methods section needs work. For example, the description of RNA extraction in l496-499 is a stub. It is not even mentioned what RNA was extracted from. Confusingly, there is a much longer version of the RNA extraction procedure on a different experiment starting in l 543. Another example is the PCA in starting in line 528. It is not even said what the subject of PCA was. Where are the details for the TF binding site enrichment analysis?

*We have now made the following changes to the different Methods parts to add more detail and thus clarity:*

*RNA extraction (lines 576-583)*

*Guts from 30 adult female flies were freshly dissected in PBS after 4 hours of infection with a pellet of Pseudomonas entomophila at OD100. The guts were then transferred to 1000uL Trizol Reagent (Invitrogen) with 10uL plastic beads, then homogenized in a Precellys 24 Tissue Homogenizer at 6000 rpm for 30 seconds. RNA extraction was performed using the manufacturer’s protocol. The RNA pellet was resuspended in 8µl of RNAse-free water prior to Nanodrop quantification and quality verification, followed by final dilution to a concentration of 500ng/ul.*

*Principal component analyses (lines 614-618)*

*The FactoMineR package was used to perform the principal component analyses on log2 count per million data as normalized by Voom after keeping expressed genes (count > 5 in more than 38 samples). PCA was performed with scaling and centering to avoid biases from differences in gene average expression or length.*

*local-eQTL analysis (lines 620-639)*

*We performed separate analyses for each infected condition with Matrix-eQTL using a linear model that accounts for genetic relatedness and Wolbachia infection status (Shabalin, 2012). Variants that are within 10kb of an expressed gene and whose minor allele frequency (MAF) is greater than 5 in the 38 tested lines were kept in the analysis. MAF here is actually the number of lines carrying the less prevalent allele in the sampled strains divided by 38. This translates to a minimum of 6/38 = 15.8%. To account for genetic relatedness, we calculated the three genotype principal components using the SNPrelate R package using a pruned set of SNPs from the DGRP freeze 2 genotypes (ld threshold = 0.2). Wolbachia infection status was obtained from the DGRP2 resource website (http://dgrp2.gnets.ncsu.edu). Associations with a p-value less than 0.001 were kept, followed by FDR estimation using the Benjamini-Hochberg procedure as implemented in Matrix-eQTL. Each gene’s expression level was transformed to a standard normal distribution based on rank. Local-eQTL associations with an FDR corrected p-value lower than 0.05 were considered significant. Metaplots were plotted in R. The GO analysis was performed using the GOstats (Falcon & Gentleman, 2007) R package (Hypergeometric test p-value < 0.005), and REVIGO (Supek, Bošnjak, Škunca, & Šmuc, 2011) was used to reduce redundancy in the ontology groups and plot them by semantic similarity (allowed similarity = 0.7). For each pair of significant GO terms, Revigo calculates Resnik’s and Lin’s semantic similarity (simRel)(Resnik, 1999). The two-dimensional representation is the result of multi-dimensional scaling (MDS) applied to the terms’ semantic similarity matrix.*

TF motif enrichment (lines 641-648)

To determine TF motif (regulatory feature) enrichment in regions around condition-specific eQTLs, we generated a BED file of the genomic coordinates of a window of 201 bases centered around each local-eQTL. We then submitted this file to i-cisTarget )(Herrmann et al., 2012; Imrichová et al., 2015) with the following settings: analysis type=Full analysis; Species = Drosophila melanogaster (dm3); database version 5.0, and all features selected. After performing the two analyses, we used the comparison tool on the website to determine differential TF motif enrichment between the infected and control-specific local-eQTL genomic regions.

Furthermore, I think the manuscript could be improved, regarding structure. If I understood correctly, there are two major transcriptome data sets: one for the eQTL analysis and one for the allele specific expression (ASE) analysis. Currently it seems to me that the manuscript alternates between them. The first results section deals with the eQTL dataset, the second with ASE, then the third comes back to the eQTL data set? Is this correct? It is not clearly mentioned what the data set is that the third results section starting at line 275 deals with. I first thought it must be the ASE data set because this section comes right after the ASE analysis and starts with PCA of gene expression. Finding out that it was probably the eQTL data set took me a while and disrupted coherence and readability. An easy solution to this might be to mention the switch back in data sets at the beginning of the paragraph.

A suggestion for the author's consideration:

A suggestion for a much more natural solution would be to start the results section with the finding from the eQTL data set that only ntc is differently expressed and then focus on how first the eQTL and then the additional ASE data were used to track down the regulatory variants. I think this approach would be logically more coherent and focused. Furthermore, I am not fully convinced that the eQTL and ASE results as such deserve the spotlight position they currently occupy. This is because there is no correlation between the results (Figure 2D and 2E) and a large proportion of loci could neither be assigned cis nor trans affects. Therefore, other parts of the manuscript are much more exciting. Although I fully respect the importance of these analyses and the work that went into it, the detailed description and interpretation of the results that seems speculative sometimes (l398-426) was distracting from the main message that I thought was that the authors provide a great example of how to establish the connection between a regulatory variant and an important phenotype. Hence, I think a focus on how eQTL and ASE data were leveraged in the context of this central theme could benefit readability and focus of manuscript.

*We thank the reviewer for making very helpful suggestions regarding how we can improve the flow of our manuscript. As indicated above in our response to reviewer 1, we have now substantially reshuffled the different parts of our manuscript such that we now present the differential gene expression analysis first, then the eQTL study followed by the allele-specific expression analysis, each time using ntc as the unifying thread throughout the manuscript for improved flow and clarity, as suggested by both reviewers.*

Minor comments:

1. l43 It is not clear what is meant by "channeled through distinct molecular layers"

*We apologize for the confusion. We meant to say “affect different molecular mechanisms” and have updated the abstract accordingly (Line 43).*

2. l79 There is a citation that was not converted to the numbering format and that is not listed in the references (Brem...).

*Thank you for pointing out this error, which is now corrected and appears as (9) (Line 79).*

3. l108 I think the choice of Pseudomonas entomophila needs some explanation for all readers not familiar with your previous work. It should be clarified at least that it is a pathogen that causes disease when ingested by the flies. Because the authors write in the discussion (l459) that they are looking an "ecologically-relevant trait" by analyzing immunocompetence towards P.e., they might also consider to mention here that Pseudomonas occurs in natural D. melanogaster population quite frequently (e.g Staubach et al 2013 or Wang & Staubach 2018).

*We have now added a small explanation rationalizing our choice of bacteria (lines 110-111).*

4. l133 It is not very well explained what is meant by individualized genomes. This should be better explained in the methods section. Does it mean you are just keeping homozygous SNPs?

*To avoid any biases in the downstream analyses, specifically in the eQTL analyses, we used the data from the DGRP 2 freeze to construct individualized genomes before aligning RNAseq reads to them. Since these strains are supposed to be inbred, any heterozygous site in the available VCF files was treated as a reference site as there is no guarantee to whether it is still heterozygous or fixed (reference or alternate) in the stocks we have in our lab. We have added more details in the methods (lines 591-601), especially regarding the motivation for using individualized genomes.*

5. l267 there is no figure 3f .

*We apologize for the typo and have now changed it to 4f (Line 368).*

6. l275 It is not "few genes" but a single gene (ntc), or am I getting something wrong?

*It is only one gene indeed and we have now changed the text accordingly (Line 128).*

7. l285-295 could go to the discussion

*We thank the reviewer for the suggestion, but we prefer keeping it in the results section as it is an analytical detail.*

8. l306-309 I do not understand.

*We have removed this analysis from the text.*

9. l313/Fig3c I would really like to see the data on the expression levels of ntc in a plot to judge the difference between susceptible and resistant lines since much of the paper hinges on this result.

*Please note that this data is now presented in Figure 2d.*

*References*

*Alpern, D., Gardeux, V., Russeil, J., Mangeat, B., Meireles-filho, A. C. A., Breysse, R., … Deplancke, B. (2019). transcriptomics enabled by bulk RNA barcoding and sequencing, 1–15.*

*Aronson, B. E., Stapleton, K. A., & Krasinski, S. D. (2014). Role of GATA factors in development, differentiation, and homeostasis of the small intestinal epithelium. American Journal of Physiology - Gastrointestinal and Liver Physiology, 306(6), 474–490. https://doi.org/10.1152/ajpgi.00119.2013*

*Buchon, N., Broderick, N. A., Poidevin, M., Pradervand, S., & Lemaitre, B. (2009). Drosophila Intestinal Response to Bacterial Infection: Activation of Host Defense and Stem Cell Proliferation. Cell Host and Microbe, 5(2), 200–211. https://doi.org/10.1016/j.chom.2009.01.003*

*Buchon, N., Osman, D., David, F. P. A., Yu Fang, H., Boquete, J. P., Deplancke, B., & Lemaitre, B. (2013). Morphological and Molecular Characterization of Adult Midgut Compartmentalization in Drosophila. Cell Reports, 3(5), 1725–1738. https://doi.org/10.1016/j.celrep.2013.04.001*

*Castel, S. E., Levy-Moonshine, A., Mohammadi, P., Banks, E., & Lappalainen, T. (2015). Tools and best practices for data processing in allelic expression analysis. Genome Biology, 16(1), 1–12. https://doi.org/10.1186/s13059-015-0762-6*

*Chakrabarti, S., Liehl, P., Buchon, N., & Lemaitre, B. (2012). Infection-induced host translational blockage inhibits immune responses and epithelial renewal in the Drosophila gut. Cell Host Microbe, 12(1), 60–70. https://doi.org/10.1016/j.chom.2012.06.001*

*Falcon, S., & Gentleman, R. (2007). Using GOstats to test gene lists for GO term association. Bioinformatics, 23(2), 257–258. https://doi.org/10.1093/bioinformatics/btl567*

*Fink, C., Hoffmann, J., Knop, M., Li, Y., Isermann, K., & Roeder, T. (2016). Intestinal FoxO signaling is required to survive oral infection in Drosophila. Mucosal Immunology, 9(4), 927–936. https://doi.org/10.1038/mi.2015.112*

*Herrmann, C., Van De Sande, B., Potier, D., & Aerts, S. (2012). i-cisTarget: An integrative genomics method for the prediction of regulatory features and cis-regulatory modules. Nucleic Acids Research, 40(15), 1–17. https://doi.org/10.1093/nar/gks543*

*Imrichová, H., Hulselmans, G., Atak, Z. K., Potier, D., & Aerts, S. (2015). I-cisTarget 2015 update: Generalized cis-regulatory enrichment analysis in human, mouse and fly. Nucleic Acids Research, 43(W1), W57–W64. https://doi.org/10.1093/nar/gkv395*

*McManus, C. J., Coolon, J. D., Duff, M. O., Eipper-Mains, J., Graveley, B. R., & Wittkopp, P. J. (2010). Regulatory divergence in Drosophila revealed by mRNA-seq. Genome Research, 20(6), 816–825. https://doi.org/10.1101/gr.102491.109*

*Pinter, S. F., Colognori, D., Beliveau, B. J., Sadreyev, R. I., Payer, B., Yildirim, E., … Lee, J. T. (2015). Allelic Imbalance Is a Prevalent and Tissue-Specific Feature of the Mouse Transcriptome. Genetics, 200(June), 537–549. https://doi.org/10.1534/genetics.115.176263*

*Resnik, P. (1999). Semantic Similarity in a Taxonomy: An Information-Based Measure and its Application to Problems of Ambiguity in Natural Language. Journal of Artificial Intelligence Research, 11, 95–130.*

*Shabalin, A. A. (2012). Matrix eQTL: Ultra fast eQTL analysis via large matrix operations. Bioinformatics, 28(10), 1353–1358. https://doi.org/10.1093/bioinformatics/bts163*

*Strober, B. J., Elorbany, R., Rhodes, K., Krishnan, N., Tayeb, K., Battle, A., & Gilad, Y. (2019). Dynamic genetic regulation of gene expression during cellular differentiation. Science, 364(6447), 1287–1290. https://doi.org/10.1126/science.aaw0040*

*Supek, F., Bošnjak, M., Škunca, N., & Šmuc, T. (2011). Revigo summarizes and visualizes long lists of gene ontology terms. PLoS ONE, 6(7). https://doi.org/10.1371/journal.pone.0021800*

**Second round of review**

**Reviewer 1**

Overall the authors have done a strong job responding to the critiques of the previous version of the manuscript.

I have three outstanding points, two of which are very minor:

(1) The author's address power - which I raised as an issue previously in the context of examining shared eQTL mapped in independent analyses (if power is low, sharing has to be low also, so "limited sharing" could be a technical, rather than a biological result) - in an indirect way by showing that regardless of the MAF of the associated variant, the level of sharing is the same. This analysis is carried out since there should be lower power to identify causative variants with lower MAF. My concern with the approach is two-fold. One is ideaological; They're not addressing power directly, but rather by examining a correlate of power (MAF). Despite the authors statement in the response, power in the DGRP is easily estimable (see the code in Box 2 of Long et al. Trends in Genetics 2014) so they can work this out at least under idealized conditions. If the value is 80% my concerns largely evaporate, but if power is 20% for the average eQTL mapped, there are genuine concerns that at least some of the overlap must be technical. Indeed, since power cannot be 100% some of the lack of overlap _must_ be technical (a failure to identify true eQTL), so at least some mention should be made of this.

Second, the assumption of the MAF analysis is that lower MAF leads to lower power, so fewer associations will be identified. They find this (e.g., red/blue lines in Supp Figure 3b), but it looks to me like the green line - which the authors argue is flat, looks like it suggests that with lower MAF there _is_ reduced overlap ob average, implying (following the authors contention) that lower power leads to lower overlap. I am comfortable with the idea that much of the reduced overlap is biological in origin, but this new analysis suggests to me that power is relevant here also.

(2) This is minor. Towards the end of the paragraph between lines 169 and 182, the authors want to make the point that expression after 4 hours doesn't clearly discriminate "resistant" from "susceptible" groups of lines, in contrast to the phenotype where (line 181) flies are "alive vs. dead". I understand where they are coming from, and understand the reasoning for the 4hr timepoint, but the flies aren't dying by 4 hours. So, regardless of the good reasons for using the 4hr timepoint, if nothing at 4 hours is predictive of death at >>4hrs after infection (and apparently RNAseq doesn't reveal anything that is predictive across all lines), it remains possible that expression at 4hrs is somewhat irrelevent to the death phenotype. I really think this deserves some acknowledgement in the work.

(3) This is also minor. On lines 199-200 the authors state that "ntc expression is strongly reduced in mutant allele lines compared to control." I made a comment about this last time which the authors addressed in the response, but the text didn't get changed, and I think clarification is useful here. In Figures 2b/c the authors show the result: ntc expression for the infected mutant -/+ flies (the control expression is impossible to determine given the y-axis employed) is essentially 1/2 that of the infected control +/+ flies. This is _exactly_ the expectation given the genotypes. So the ntc expression test here is solely being done to confirm that the ntc mutant is indeed a null. I think all the authors need to do is acknowlege on lines 199-200 that this result is entirely expected.

**Authors’ response to reviewers**

**Reviewer 1**

• (Power analysis) The author's address power - which I raised as an issue previously in the context of examining shared eQTL mapped in independent analyses (if power is low, sharing has to be low also, so "limited sharing" could be a technical, rather than a biological result) - in an indirect way by showing that regardless of the MAF of the associated variant, the level of sharing is the same. This analysis is carried out since there should be lower power to identify causative variants with lower MAF. My concern with the approach is two-fold. One is ideaological; They're not addressing power directly, but rather by examining a correlate of power (MAF). Despite the authors statement in the response, power in the DGRP is easily estimable (see the code in Box 2 of Long et al. Trends in Genetics 2014) so they can work this out at least under idealized conditions. If the value is 80% my concerns largely evaporate, but if power is 20% for the average eQTL mapped, there are genuine concerns that at least some of the overlap must be technical. Indeed, since power cannot be 100% some of the lack of overlap _must_ be technical (a failure to identify true eQTL), so at least some mention should be made of this.

Second, the assumption of the MAF analysis is that lower MAF leads to lower power, so fewer associations will be identified. They find this (e.g., red/blue lines in Supp Figure 3b), but it looks to me like the green line - which the authors argue is flat, looks like it suggests that with lower MAF there _is_ reduced overlap ob average, implying (following the authors contention) that lower power leads to lower overlap. I am comfortable with the idea that much of the reduced overlap is biological in origin, but this new analysis suggests to me that power is relevant here also.

*Response:*

*We thank the reviewer for his/her suggestion. We have followed the reviewer’s recommendation to calculate power according to Long et al. (TIG, 2014). For that, we determined power over a wide range of minor allele frequencies (MAF) and genetic contributions to gene expression variance, aiming to cover the range of these two parameters in our data, which we also report in Additional file 1: Fig S3. These analyses revealed that power is indeed low for some of our local-eQTLs, notably those that have a low MAF and low genetic contribution. However, we also found that we have a uniform representation of local-eQTLs for all MAFs and a distribution of genetic contributions that peaks between 40% and 50%, with simulated power increasing from ~20% to ~75%. In addition, we are very well powered (>80%) to detect true local-eQTLs that contribute to 60% of the gene expression variance when MAF > 0.3. Given this continuum of power that ultimately depends on the MAF and the effect of a local-eQTL on gene expression, and the increasing ability to detect true local-eQTLs, and consequently true shared local-eQTLs, one would expect increased sharing of local-eQTLs with increased power. However, our actual data do not support this, suggesting instead that shared local-eQTLs are indeed depleted. The manuscript and figures have been adapted to clarify this. Namely:*

*- We have added new panels in Fig S3 that show the characterization of the obtained local-eQTLs in terms of MAF and genetic contribution to variance.*

*- We have performed a new simulation study based on the code provided by Long et al., 2014.*

*- We have acknowledged our low power to detect an eQTL when MAF is low in the text, in addition to describing the rationale behind and interpretation of this new power analysis.*

*Here are the text added to the results and the discussion:*

*Results*

*To address this possibility, we first characterized the allele frequency spectrum of all significant local-eQTLs and found no systematic bias in allele frequency with respect to the number of identified local-eQTLs (Additional file 1: Fig. S3a). For each local-eQTL, we then calculated the percentage of variance explained by genotype, and again found no clear relationship between allele frequency and the number of shared local-eQTLs (Additional file 1: Fig. S3b). Subsequently, we performed simulations under idealized conditions for a wide range of allele frequencies and genetic contributions to variance, following a strategy described in (50) (Additional file 1: Fig. S3c). These analyses revealed that the power to detect a local-eQTL has a broad range, implying that many condition-specific and shared local-eQTLs are likely not detected in our study at the lower bounds of the allele spectrum or genetic contribution. For example, the power to detect a local-eQTL with a MAF of 0.15 and a genetic contribution of 30% is 10% whereas it reaches 100% when the MAF is 0.5 and genetic contribution is 50%. We also simulated the power of detecting a shared local-eQTL by performing simulations in pairs that share the same genetic components but with a random environmental component. As expected, the power to identify a shared local-eQTL increases sharply as a function of increasing allele frequency and genetic contribution to trait variance (Additional file 1: Fig. S3c). These simulations imply that if limited power leads to more condition-specific local-eQTLs, we should observe a relatively greater number of condition-specific local-eQTLs in the lower allele-frequency spectrum. However, we did not observe such a trend in our actual data. In fact, the odds of identifying condition-specific local-eQTLs versus shared local-eQTLs did not change as a function of allele frequency (Additional file 1: Fig. S3d-f). Given these observations, we conclude that limited power cannot be a major reason for the observed, low number of shared local-eQTLs.*

*Discussion*

*However, the majority of local-eQTLs proved to be condition-specific, including the local-eQTLs at the ntc locus. Since we demonstrate that limited power cannot adequately explain this observation, our data suggest that cryptic variation has an important contribution to infection resistance (74,75)*

• (Minor) Towards the end of the paragraph between lines 169 and 182, the authors want to make the point that expression after 4 hours doesn't clearly discriminate "resistant" from "susceptible" groups of lines, in contrast to the phenotype where (line 181) flies are "alive vs. dead". I understand where they are coming from, and understand the reasoning for the 4hr timepoint, but the flies aren't dying by 4 hours. So, regardless of the good reasons for using the 4hr timepoint, if nothing at 4 hours is predictive of death at >>4hrs after infection (and apparently RNAseq doesn't reveal anything that is predictive across all lines), it remains possible that expression at 4hrs is somewhat irrelevent to the death phenotype. I really think this deserves some acknowledgement in the work.

*Response: To acknowledge this, we have revised this paragraph as follows: “This observation supports the notion that the differences between the classes, while being overt at the physiological level (i.e. being alive vs. dead), cannot be fully explained at the single gene level using standard differential expression approaches, at least at the sampled 4h post-infection time point.”*

• (Minor) On lines 199-200 the authors state that "ntc expression is strongly reduced in mutant allele lines compared to control." I made a comment about this last time which the authors addressed in the response, but the text didn't get changed, and I think clarification is useful here. In Figures 2b/c the authors show the result: ntc expression for the infected mutant -/+ flies (the control expression is impossible to determine given the y-axis employed) is essentially 1/2 that of the infected control +/+ flies. This is _exactly_ the expectation given the genotypes. So the ntc expression test here is solely being done to confirm that the ntc mutant is indeed a null. I think all the authors need to do is acknowlege on lines 199-200 that this result is entirely expected.

*Response: Thank you for allowing us to clarify: “Furthermore, we performed RT-qPCR on dissected guts from the lines crossed to bw;st and found that ntc expression is, as expected, strongly reduced in mutant allele lines compared to control.”*
